# Step-by-step guide to efficient subtomogram averaging of virus-like particles with *Dynamo*

**DOI:** 10.1371/journal.pbio.3001318

**Published:** 2021-08-26

**Authors:** Stefano Scaramuzza, Daniel Castaño-Díez

**Affiliations:** BioEM Lab, Biozentrum, University of Basel, Basel, Switzerland; California Institute of Technology, UNITED STATES

## Abstract

Subtomogram averaging (STA) is a powerful image processing technique in electron tomography used to determine the 3D structure of macromolecular complexes in their native environments. It is a fast growing technique with increasing importance in structural biology. The computational aspect of STA is very complex and depends on a large number of variables. We noticed a lack of detailed guides for STA processing. Also, current publications in this field often lack a documentation that is practical enough to reproduce the results with reasonable effort, which is necessary for the scientific community to grow. We therefore provide a complete, detailed, and fully reproducible processing protocol that covers all aspects of particle picking and particle alignment in STA. The command line–based workflow is fully based on the popular *Dynamo* software for STA. Within this workflow, we also demonstrate how large parts of the processing pipeline can be streamlined and automatized for increased throughput. This protocol is aimed at users on all levels. It can be used for training purposes, or it can serve as basis to design user-specific projects by taking advantage of the flexibility of *Dynamo* by modifying and expanding the given pipeline. The protocol is successfully validated using the Electron Microscopy Public Image Archive (EMPIAR) database entry 10164 from immature HIV-1 virus-like particles (VLPs) that describe a geometry often seen in electron tomography.

## Abstract 2

This study presents a complete and detailed step-by-step guide for subtomogram averaging using Dynamo software, with a special focus on particle picking and particle averaging; this will enable efficient processing for all experience levels, and lays a foundation for user-specific projects.

## Introduction

Cellular organelles and biological macromolecules such as proteins and complexes thereof play a fundamental role in almost all life sciences. In structural biology, the molecular structure of these particles is studied to gain information about their morphology and function. Electron tomography is a well established and quickly evolving technique that, additionally to determining the 3D structure of the particles of interest, also allows to image the particles in situ, and, therefore, to draw conclusions about their cellular context, geometry, and interactions with their environment.

A powerful image analysis technique in electron tomography is subtomogram averaging (STA), where copies of the same particle of interest within a tomogram are extracted independently and then aligned and averaged to a common reference in order to increase the signal and detail of the underlying structure. STA has led to many breakthroughs in structural biology, and method development is ongoing [1–5]. A big challenge in STA is the high complexity of the technique, caused by the often intricate geometries of the biological structures that often show large variations between projects. This makes tasks such as locating particles (particle picking) within the tomograms particularly difficult.

Various software for STA exist. Among the popular ones are *Dynamo* [6,7], TOM [8], AV3 [9], PyTOM [10,11], EM-Clarity [12], RELION [13], EMAN2 [14], PEET [15,16], M [17], and MLTOMO [18]. Guides and tutorials on how to use these software packages can be found on the corresponding websites. For *Dynamo* and RELION, there are published processing protocols covering specific parts of the processing pipeline [13,19].

Published structures in STA are often difficult to reproduce due to the lack of in-depth information on the methods, since providing this information is usually beyond the scope of such publications. To date, only a few protocols and tutorials are available that go deeply into the practical aspects of STA processing [13,19]. Our experience in teaching STA showed that while users often have a good grasp on the theory, they often struggle with exactly those practical details that are rarely available. We want to meet the need for such information and therefore created this protocol that is intended to provide a complete, detailed, and fully reproducible step-by-step guide for particle picking and particle alignment in *Dynamo*. The script-based approach shows how the *Dynamo* tools can be combined with MATLAB scripts to create a straightforward, versatile, and ready-to-use solution. The shown pipeline can also serve as a basis for user-specific projects, since it can be extended or adapted to other geometries such as, e.g., lipid tubes or other types of surfaces. We also demonstrate how parts of the processing pipeline can be streamlined and automated to improve productivity. The protocol is aimed at users of all levels and can be used for training or as a guide to set up user-specific projects.

The *Dynamo* software for STA, which is written mainly in MATLAB (MathWorks (www.mathworks.com)), was chosen for this report because of its popularity and versatility. Functions that independently address all steps needed in the STA processing can be individually called and combined with conventional MATLAB scripts, making the software very flexible and allowing to set up customizable processing pipelines with high levels of automation. This versatility is essential for STA because it allows to design and adapt image processing strategies dependent on the often unique geometries of the analyzed samples.

In this protocol, we process the Electron Microscopy Public Image Archive (EMPIAR) dataset with the ID 10164 (related Protein Data Bank (PDB) entry 5l93) from immature HIV-1 virus-like particles (VLPs) [20] using the 5 tomograms that were used in [21]. We chose this dataset because it has been already used for benchmarking in various other STA projects [12]. More importantly, the sample geometry in this specific dataset consists of particles on the surface of a sphere, which is a geometry often seen in electron tomography. The same protocol can therefore easily be adapted to any similar samples such as, e.g., membrane proteins reconstituted in lipid vesicles or any other type of spherical viruses.

The emphasis of the protocol is on particle picking and particle alignment in *Dynamo*. Nevertheless, the pre- and postprocessing steps are briefly explained to ensure full reproducibility. In an effort to reduce the number of variables for the users, we limited the dependency on third-party software in those steps by using simple 2D contrast transfer function (CTF) correction of the tomograms. The way we perform pre- and postprocessing is one of many ways to do it right, and users are free to use their preferred software for those steps. The protocol can be summarized in the following 3 parts:

1. Preprocessing: This part covers drift correction, dose weighting, CTF correction, tilt series alignment, and tomogram reconstruction.
2. STA: The core of this protocol paper is a command line–based workflow (divided in 4 processing scripts). It is written in MATLAB and includes *Dynamo* functions. It covers all steps from particle picking to gold standard refinement, and it is designed for minimal user intervention.
3. Postprocessing: This part covers the resolution estimation, sharpening, and filtering.

## Materials and equipment

### Hardware requirements

The following hardware components with the recommended minimal specifications are needed:

- a computer or workstation;
- sufficient disk storage (>3 TB) and random access memory (RAM, >64 GB);
- access to multiple graphic processing units (GPUs) for subtomogram alignment. We suggest a minimum of 2 state-of-the-art GPUs (e.g., NVIDIA GeForce RTX 2080 Ti) but recommend at least 6, since subvolume alignment is the most computationally intensive step in the processing; and
- access to a multicore computing environment for the averaging of subtomograms (>12 processing cores).

### Software requirements

**Operating system**

- Linux (recommended) or macOS. Windows was not tested, and extra steps may be necessary to ensure compatibility.

**Preprocessing**

- MotionCor2 [22] for drift correction;
- CTFFIND4 [23] for defocus estimation; and
- IMOD [24] for tilt series alignment and tomogram reconstruction.

**Subtomogram averaging**

- MATLAB (Mathworks) version R2019a or newer. For users unfamiliar with MATLAB or similar coding languages, we highly recommend to learn some of its basics. Users should ideally be familiar with terms such as arrays, loops, and functions.
- *Dynamo* software for STA (version 1.1.520 or newer). A stand-alone version of *Dynamo* exists that works independently of MATLAB. For this protocol, however, we recommend to use the MATLAB version. The download links, extensive documentation, and guides can be found online (www.dynamo-em.org).
- Chimera UCSF [25] for subtomogram annotations.

**Postprocessing**

- RELION 3 [26] for resolution estimation and sharpening; and
- Bsoft [27] for local resolution estimation (function blocres).

### Dataset

From the EMPIAR entry 10164 (download here: www.ebi.ac.uk/pdbe/emdb/empiar/entry/10164), the frames of the 5 tilt series according to Table 1 are used.

**Table 1:** Dataset. Tomogram name and dose rate of the 5 tomograms from the EMPIAR-10164 dataset that are used in the processing.

Name	Dose (e^−^/Å/s)
TS_01	3.0
TS_03	3.0
TS_43	3.1
TS_45	3.1
TS_54	3.0

EMPIAR, Electron Microscopy Public Image Archive.

The frames were recorded on a FEI Titan Krios transmission electron microscope (spherical aberration: 2.7 mm) operated at 300 keV equipped with a Gatan K2 direct electron detector using the dose-symmetric tilt scheme [28]. The calibrated 4K pixel size is 1.35 angstrom. More details about data acquisition is found in the Supporting information of the corresponding publication [20].

## Procedure

### Preprocessing

Drift correction of the frames and Fourier binning [29] to 3710×3838 pixels is done using MotionCor2. The used parameters are listed in Table 2. This step can be done using the *Dynamo* wrapper dpcomp.motioncor.wrapper. The *Dynamo* tool dpktut.hiv.preprocess can be used to create *mrc* stacks from the individual micrographs.

**Table 2:** Preprocessing parameters. Parameters used in MotionCor2 and CTFFIND4.

**MotionCor2**	
parameter	value
patch iterations B-factor tolerance binning	5×5 30 200 0.5 2
**CTFFIND4**	
parameter	value
pixelsize amplitude spectrum size resolution range defocus range defocus search step astigmatism restraint	1.35 Å 512 10–50 Å 10,000–60,000 Å 10 Å 30 Å

Defocus estimation is done using CTFFIND4 on each tilt. For defocus estimation, we consider only the micrograph area that corresponds to the projection of the tilted micrograph from 0° tilt. This angle-dependent cropping leads to a more robust defocus estimation. The parameters used in CTFFIND4 are listed in Table 2. This step can be done using the wrapper dpcomp.ctffind.wrapper.

Dose weighting is done after defocus estimation on each tilt using a MATLAB implementation of the algorithm introduced in Unblur [30] and using the accumulated dose corresponding to the last frame of each tilt (reduced by 20% to be more conservative). This step can be done using the *Dynamo* wrapper dpktilt.filters.exposure. Alternatively, the option for dose weighting implemented in IMOD can be used.

Tilt series alignment and tomogram reconstruction are done in IMOD. The seed model for the gold fiducials is first generated automatically and then completed manually in order to obtain about 8 to 16 bead tracks per tilt series. Fiducials are tracked automatically, and gaps are fixed manually. Fine alignment is done by estimating only 1 rotation and keeping all other parameters fixed. Residuals are minimized according to our online guide (http://www.dynamo-em.org/w/index.php/Considerations_for_tilt_series_alignment_in_IMOD). Residuals should be optimized until a root mean square (RMS) of below 2 pixels is achieved. Defocus estimation in IMOD is skipped, and the results from CTFFIND4 are used instead. The CTFFIND4 output file can be transformed into an IMOD compatible defocus file using the tool dpcomp.ctffind.forImod. CTF correction is done using phase flipping. During tomogram positioning, make sure the tomogram is thick enough to include the whole sample. Also, the x-axis tilt and angle offset should remain at zero. This ensures that the z-axis of the tomogram coincides with the electron beam direction, facilitating the interpretation of the volumes (e.g., geometry of the missing wedge). Finally, only 1 full-sized tomogram is generated for each tilt series using weighted backprojection (WBP). Any necessary binning will be done later on the fly directly on the subvolumes themselves during their alignment.

The final tomograms should be named after the *Dynamo* file naming convention (www.dynamo-em.org/w/index.php/Practical_Suggestions_for_Tomographic_Reconstruction). Here, we expect to have the 5 following tomograms at the end of the processing:

b001ts001.rec

b001ts003.rec

b001ts043.rec

b001ts045.rec

b001ts054.rec

For users that want to skip the preprocessing, these tomograms can be found on EMPIAR (EMPIAR-10702).

### Preparing data structure for processing scripts

Before running the processing scripts, a consistent data structure needs to be set up. This is best done by following the *Dynamo* convention as explained in the next paragraphs.

First, the 3 following directories should be created within the main project directory:

- catalogues: contains files related to the *Dynamo catalogue* including the *doc* and *vll* files;
- particles: contains the various particle folders that will be created during the processing; and
- projects: contains the processing script itself, all alignment projects, and individual files (e.g., masks) that are generated during processing.

Next, a *volume list* or *vll* file that contains the path (absolute or relative) to the tomograms needs to be created and stored in the catalogues folder (it will be used to load all tomograms into the *Dynamo catalogue* at once). Create a text file named tomograms.vll with the following content:

/path_to_tomogram/b001ts001.rec

/path_to_tomogram/b001ts003.rec

/path_to_tomogram/b001ts043.rec

/path_to_tomogram/b001ts045.rec

/path_to_tomogram/b001ts054.rec

Then, a *doc* file that also contains the path (absolute or relative) to the tomograms and their volume ID needs to be created and also stored in the catalogues folder. This file will be used to extract subvolumes from the tomograms. Create a text file named tomograms.doc with the following content:

1 /path_to_tomogram/b001ts001.rec

2 /path_to_tomogram/b001ts003.rec

3 /path_to_tomogram/b001ts043.rec

4 /path_to_tomogram/b001ts045.rec

5 /path_to_tomogram/b001ts054.rec

The processing scripts (setup.m, oversample.m, locate.m and refine.m) need to be saved into the folder projects. The scripts can be found in the Supporting information S1 Appendix, or they can be retrieved directly from Dynamo (where they are maintained and updated with every new version) using the commands dpktut.hiv.setup, dpktut.hiv.oversample, dpktut.hiv.locate, and dpktut.hiv.refine.

Finally, the *Dynamo* catalogue has to be set up by first opening MATLAB and then loading *Dynamo*. After navigating to the catalogue folder, the catalogue manager can be loaded with the command dcm. Details on how to use the catalogue can be found in our online guide (www.dynamo-em.org/w/index.php/Dcm_GUI). A new catalogue should be created and named c001. The tomograms can then be imported by loading the previously created file tomograms.vll. Afterwards, the tomograms need to be binned by first selecting all volumes and then clicking *create binned version* from the menu (use factor 2). This binned volumes are only used for *Dynamo* internal purposes and not for the actual processing.

### Description of processing script

The 4 processing scripts form the core of this protocol. They include all processing steps starting from particle picking to generating the 2 gold standard half-maps of the structure. They are completely written in MATLAB and *Dynamo*. The scripts are fully automated and are run in a sequential order, either by typing their filename (without extension) in the MATLAB command line and pressing the *enter* key or by opening them in the MATLAB editor and clicking the button *run*. User interaction is minimized, and it is only required between the execution of the scripts for tomogram and subtomogram annotations.

**Fig 1:**
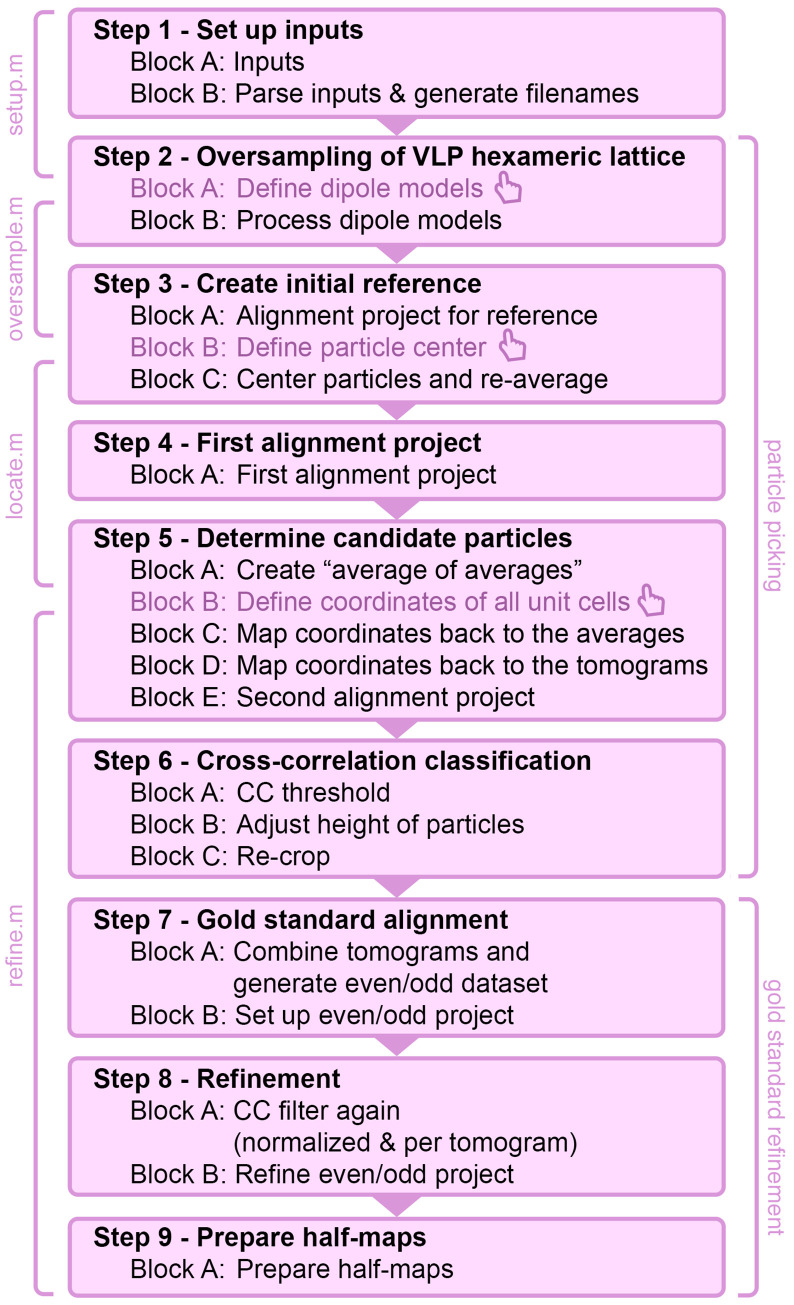
Flowchart of processing scripts. Overview of all steps and blocks from the corresponding processing scripts. All steps are automated apart from the tomogram and subtomogram annotations (marked with the hand symbol) that are required between the execution of the individual scripts. The 2 main processing categories (particle picking and gold standard refinement) are highlighted. CC, cross-correlation; VLP, virus-like particle.

The scripts are structured in a total of 9 main processing steps that are further divided into so-called blocks (see flowchart in Fig 1). In this report, the functionality of each step is explained in a dedicated section. Each section contains in the beginning a general description of the corresponding step, followed by a detailed explanation of each block. Details about single commands are further commented directly in the code of the scripts themselves. More information about the used commands can also be found using the command help for MATLAB functions or dhelp for *Dynamo* functions.

The scripts are designed in a way that allows to pause and resume processing after every step. Only setup.m that loads the global inputs has to be run once every time MATLAB is restarted.

The scripts are designed to set up and run alignment projects locally in the directory projects. To run the alignment projects in another location (e.g., on a cluster), users should use the dvtar command instead of dvrun and follow the corresponding online guidelines for transferring of alignment projects (www.dynamo-em.org/w/index.php/Tarring_projects).

Before starting with the first step of the processing, it should be verified that all processing scripts are located in the folder projects. To be able to run the scripts, the current folder in MATLAB should be the projects folder as well.

An overview of the approximate processing times is given in S1 Table. An overview of all intermediate files used during processing is given in S2 Table. The listed files (including the tomograms) are also available on EMPIAR (EMPIAR-10702) and can be used to skip individual processing steps. The data and code to reproduce all shown figures are as well provided.

### Step 1: Set up inputs

#### Description

Inputs that will be used throughout the script are defined in this step. This includes filenames, directory paths, and processing parameters.

#### Block A: Inputs

User-specific inputs that may need to be adapted to the user environment. The path to the *Dynamo catalogue*, *doc*-file, and particle folder may be entered relative to the scripts location or absolute. The geometry-related parameters are optimized for this dataset and should not be adapted, unless another type of dataset is used. The reasoning behind the values of these parameters is explained in the block where the parameters are used for the first time. Computation related inputs need to match the users’ hardware setup.

#### Block B: Parse inputs and generate filenames

This block contains filenames that are automatically generated or derived from the previous inputs. These do not need to be adapted.

### Step 2: Oversampling of VLP hexameric lattice

#### Description

In this step, the surface of the VLPs is oversampled, and the first set of subvolumes is extracted. The goal of oversampling is to extract enough overlapping subvolumes such that every unit cell of the hexameric lattice (i.e., every 18-meric assembly of the capsid protein p24) that forms the VLPs has the chance to be present in at least 1 subvolume. In this process, also initial orientations normal to the VLP surface are imposed on the subvolumes.

#### Block A: Define dipole models

To generate the surface parametrization of the VLPs (segmentation), only their center and radius need to be manually defined. This is done using the *dipole models* from *Dynamo*. For this, the tomograms are opened through the *catalogue* in *dtmslice* (make sure to open the previously binned versions). A *dipole model set* is then opened, and every visible VLP is marked with only 2 clicks: one on its center and one on its surface. Pressing *enter* saves the current dipole in the set and activates the next dipole. This annotation does not need to be very accurate at this point, since the following alignment projects are designed to cope with inaccuracies introduced at this step (inaccuracies up to 40 pixels are tolerable). Also, VLPs with defects should be annotated, since bad/junk particles will be excluded in a dedicated step later on. About 8 to 9 VLPs per tomogram should be marked. More details about the creation of *dipole set* models are shown in the online guide (www.dynamo-em.org/w/index.php/Walkthrough_for_lattices_on_vesicles).

#### Block B: Process dipole models

**Fig 2:**
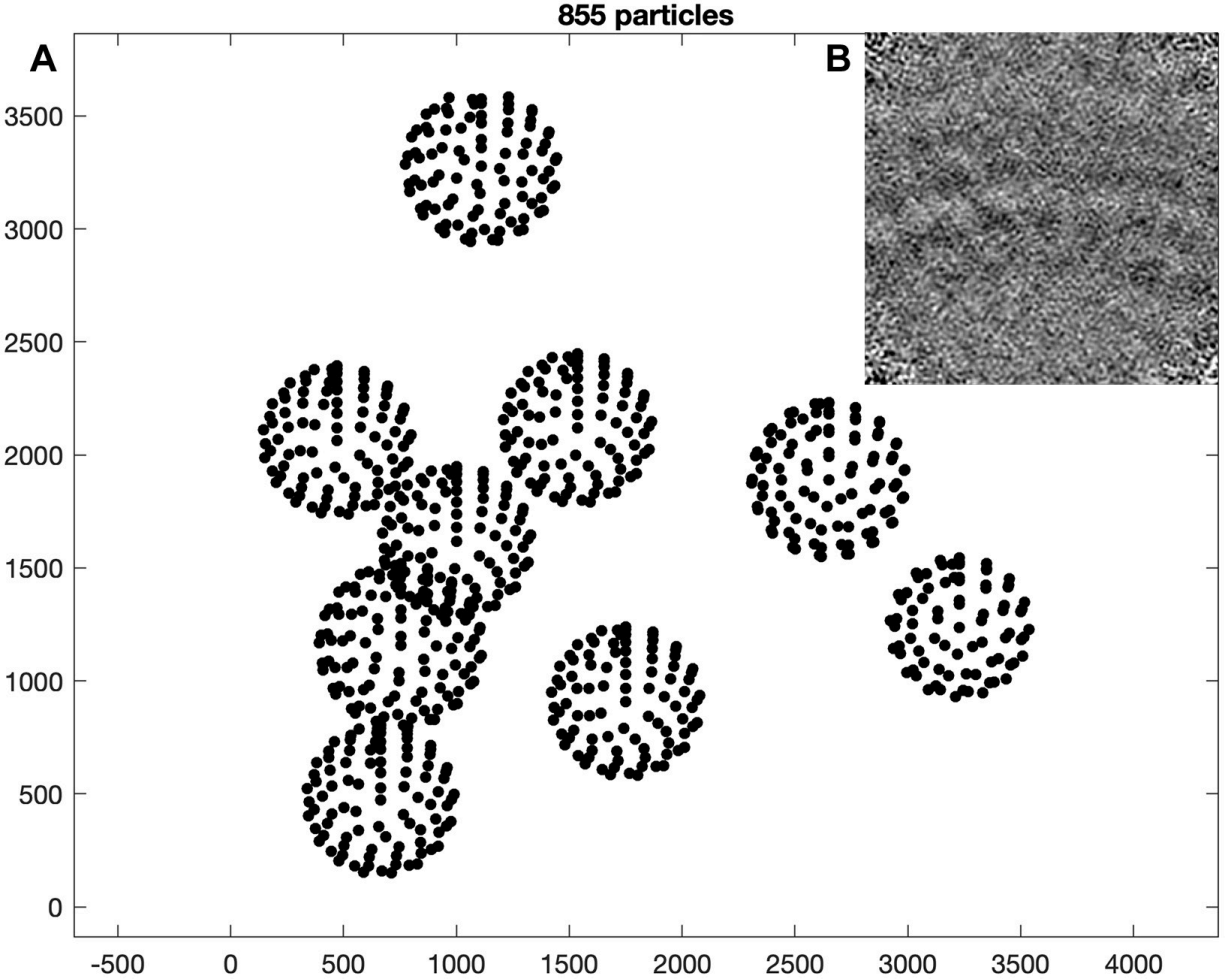
Oversampling of VLPs and average. **(A)** Crop points of tomogram *ts001* (z-view) visualized with the *Dynamo* command dtplot. The crop points are located with a regular spacing on the surface of spheres. **(B)** Average of cropped particles (projected in x-view). The curvature of the VLP surface is visible. The data and commands to exactly reproduce this figure are available on EMPIAR (EMPIAR-10702). EMPIAR, Electron Microscopy Public Image Archive; VLP, virus-like particle.

Each dipole is processed by running the so-called *model workflow* to create a regular lattice of coordinates (about 800 to 1,000 per tomogram) on the surface (see Fig 2A). These coordinates, or crop points, will be the center of the extracted (or cropped) subvolumes. The oversampling is achieved by setting the spacing (or separation) between the crop points to 120 pixels, which is a bit less than half the side length (or box size) of the cropped subvolumes (256 pixels). This side length is large enough to fit multiple unit cells of the lattice. Coordinates of the individual unit cells will be identified later. For each tomogram, the subvolumes are extracted and stored in a tomogram-specific directory named pa_ts???_s256, where the question marks are replaced by the tomogram number. A simple average without alignment should already reveal the curvature of the surface (see Fig 2B).

### Step 3: Create initial reference

#### Description

The initial reference that will be used for later projects is generated. This is done by aligning the particles from tomogram b001ts001.rec and by readjusting the center of the resulting average.

#### Block A: Alignment project for reference

First, a template for the alignment project is generated by averaging the particles from tomogram b001ts001.rec with a randomized azimuth angle. This randomization is necessary to prevent systematic errors caused by the missing wedge. The alignment project is then set up using *Dynamo* commands. This is traditionally done manually via the *dcp* graphical user interface (GUI), but, here, the aim is to minimize user intervention. The alignment project is created with the command dcp.new and by passing the inputs for the data folder, the table, and the template. After that, the project parameters are defined. The project consists of 2 rounds (or parameters sets), with 3 iterations each. The angular search range and the allowed shifts are initially rather large, since this is the first alignment done on the particles. In the second round, the search range is reduced. To speed up the processing, subvolumes are binned on the fly by defining their side length (parameter dim). In the first round, they are binned twice, and in the second round, once. There is no symmetry imposed to avoid any initial bias. The low-pass filter is set at 23 Fourier pixels (corresponding to 15 Å based on the side length of 256 pixels and pixel size of 1.35 Å). If a stronger low-pass filter is preferred, an additional round can be added in the beginning. Here, we start with 15 Å already to reduce the processing time for this protocol. A description of all alignment parameters is given in the script. After running the project, an additional copy of the resulting average is saved with inverted contrast and strong low-pass filter. This copy will be used later for visualization purposes in Chimera USCF.

#### Block B: Define particle center

**Fig 3:**
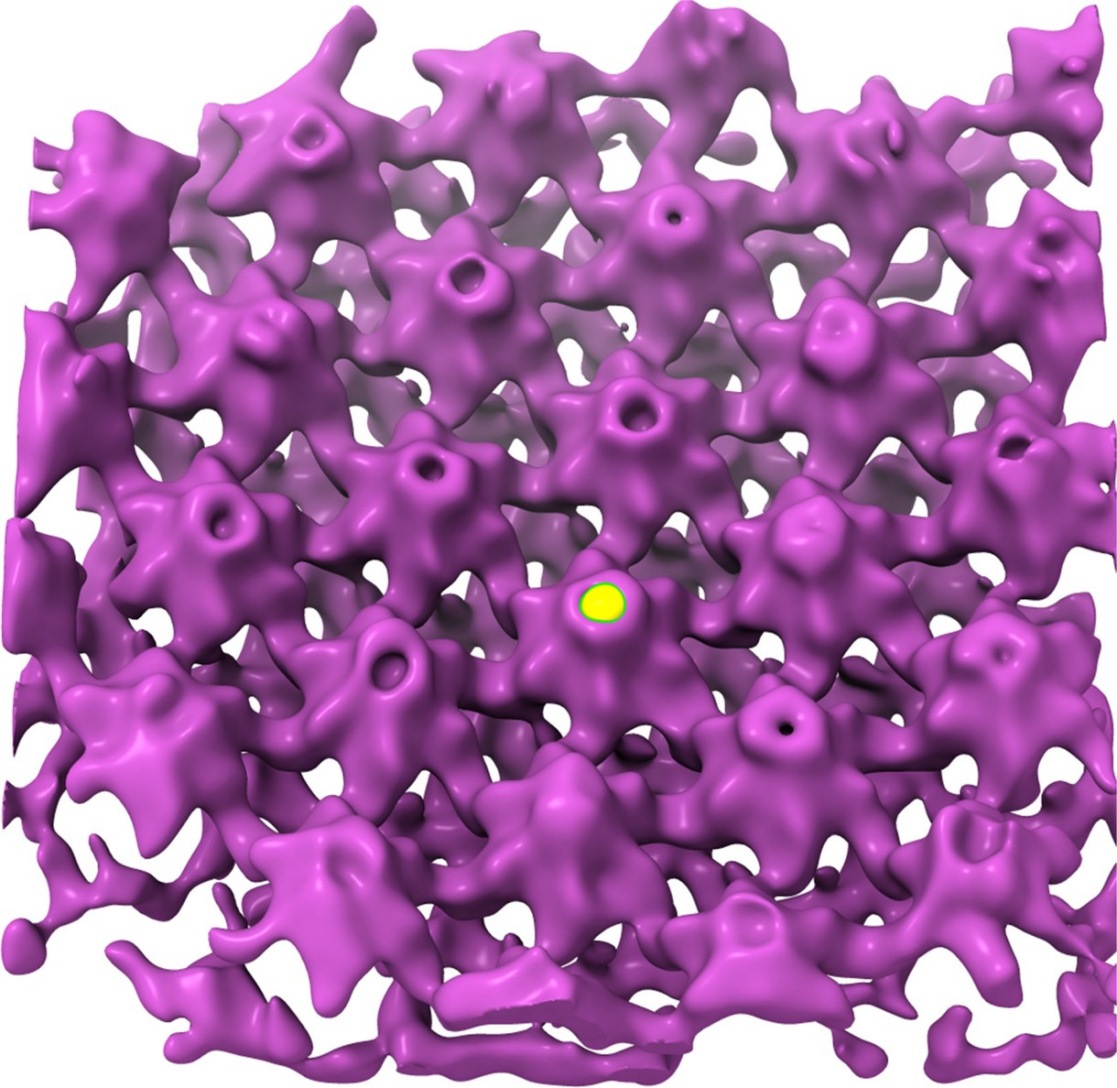
Center of unit cell defined in Chimera UCSF. The center of a unit cell (yellow dot) was marked by first rotating the average upside down and then marking the tip of the CA-N terminal domain using the *volume tracer* tool.

The previously saved average with the filename result_pr_ts001_0_INVERTED.em is opened in Chimera USCF. Using the *volume tracer* tool, the center of the most central unit is marked as shown in Fig 3. The coordinate is then stored by saving the *current marker set* with the filename reference_center.cmm.

#### Block C: Center particles and re-average

The resulting average from the alignment project is adjusted such that the center of the previously marked unit cell matches the center of the subvolume. This is achieved by first transforming the coordinates of all particles in the table using the previously defined center coordinate and then re-averaging all subvolumes. The resulting average will be the starting reference for the next alignment projects.

### Step 4: First alignment project

#### Description

In this step, all subvolumes from each tomogram are aligned separately to the previously created reference. The resulting tomogram averages will form the basis for the particle picking, where the coordinates of the individual lattice units will be determined.

#### Block A: First alignment project

**Fig 4:**
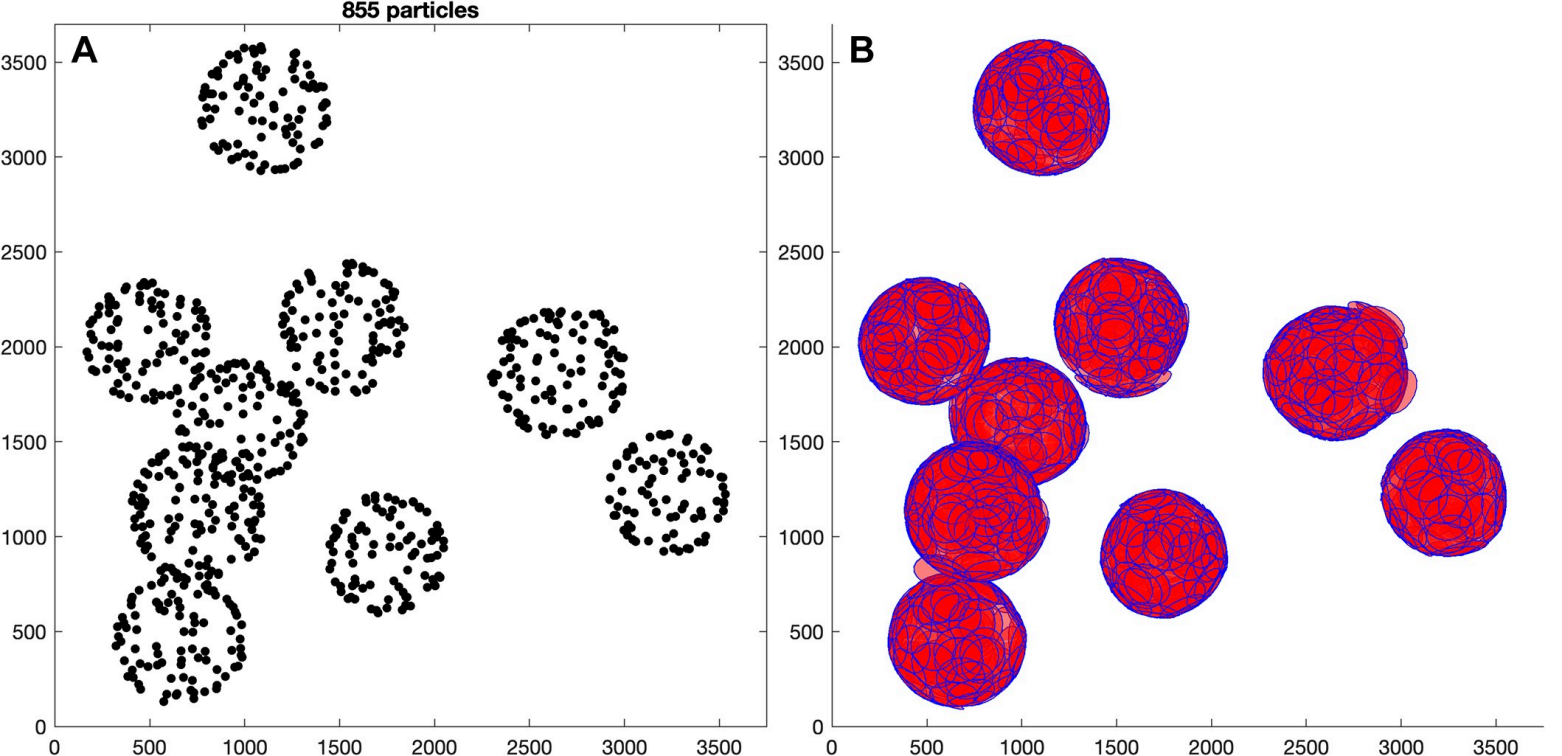
Results of first alignment. The left plot shows the coordinates of tomogram *ts001* (z-view) visualized with the *Dynamo* command dtplot using the resulting table from the first alignment. The coordinates moved from their initial positions (compare Fig 2) and adapted to the shape of the VLPs. The cropped subvolumes are large enough to cover all the empty spaces between the coordinates. This is visualized in the plot on the right where disks with a diameter matching the side length of the subvolumes (256 pixels) are placed at each coordinate and successfully cover the whole surface of the VLPs (visualized using the *Dynamo* command dpktbl.plots.disks). The data and commands to exactly reproduce this figure are available on EMPIAR (EMPIAR-10702). EMPIAR, Electron Microscopy Public Image Archive; VLP, virus-like particle.

Using a loop over all tomograms, the particles of each tomogram are aligned to the previously created reference. The azimuth of the subvolumes is randomized again to minimize potential missing wedge artifacts. The alignment parameters are identical to the previous reference project, with the only difference that from now on a C6 symmetry is imposed. The resulting coordinates (see Fig 4) are expected to have moved away from their initial positions (compare Fig 2) and to have adapted to the shape of the VLPs, which is not strictly spherical.

### Step 5: Determine candidate particles

#### Description

**Fig 5:**
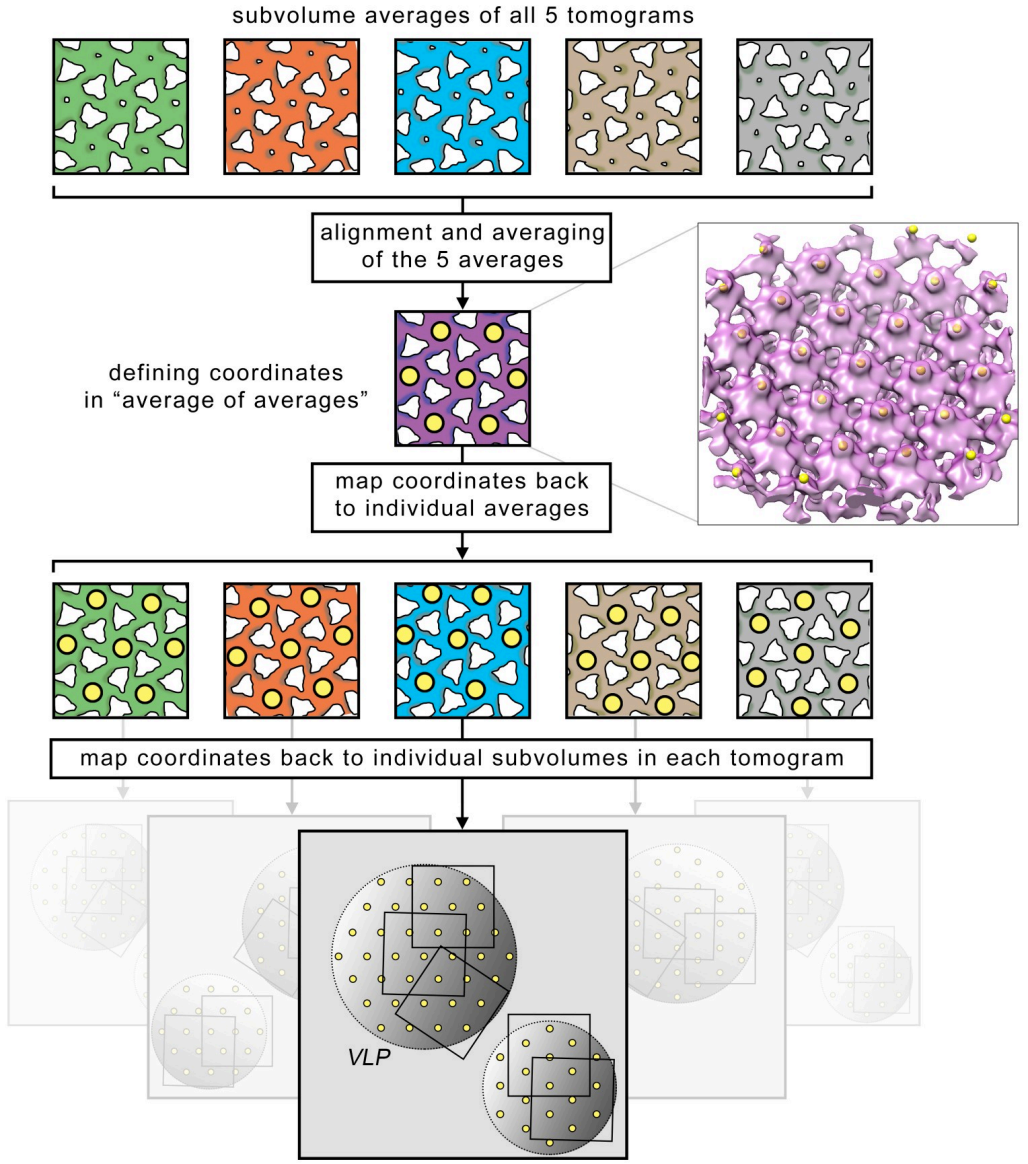
Illustration of 2-step subboxing for particle picking. After the tomogram averages have been aligned and the “average of averages” has been computed, the coordinates (yellow dots) of all unit cells are manually marked in the “average of averages.” The coordinates are then first mapped onto the tomogram averages, and, finally, onto the corresponding tomograms themselves, resulting in a coordinate grid with the expected hexameric geometry fully covering the surface of the VLPs. On the right of the illustrated “average of averages,” all marked centers of the unit cells on the actual “average of averages” produced with this protocol are shown. The coordinates were marked in Chimera UCSF using the *volume tracer* tool. With this procedure, candidate coordinates of all unit cells can be determined for the full dataset by manually labeling only 1 single density map. VLP, virus-like particle.

The aim of particle picking is to determine the coordinate of every unit cell of the hexameric lattice (or every 18-meric assembly of the capsid protein p24). In this step, the first part of particle picking is done, in which all candidate coordinates are determined (false coordinates will be excluded in the following step). Using the results from the previous alignment projects, the candidate coordinates will be determined using a 2-step subboxing procedure (in subboxing, coordinates of a specific structural feature are first defined in an average. Since the average is composed by multiple subvolumes with individual orientations, these coordinates can then be translated (or mapped) onto each of those subvolumes, and, finally, also onto the tomograms themselves. With this method, positions of features only visible in an average can be annotated in the full tomograms. See also the online guide: www.dynamo-em.org/w/index.php/Advanced_starters_guide#Subboxing). In principle, one subboxing step would suffice, in which all unit cells in each tomogram average are marked and mapped back into the corresponding tomogram. However, this would have to be done for each tomogram individually. To avoid the extra labor, an additional subboxing step is introduced, in which all tomogram averages are first aligned to each other to form an “average of averages.” All unit cells visible in this “average of averages” are marked only once and then mapped back to the tomogram averages in a first step. In the second step, they are then mapped from the averages onto the tomograms, leading to a new set of coordinates. The process is illustrated in Fig 5. Using these new coordinates, new subvolumes are extracted and aligned.

#### Block A: Create “average of averages”

The “average of averages” is created by setting up and running a small alignment project and treating the tomogram averages as input data. The corresponding data folder is created by copying the tomogram averages into it and by renaming them following the *Dynamo* convention for particle filenames (www.dynamo-em.org/w/index.php/Data_folder). Here, each particle tag number corresponds to the tomogram number. A minimal *Dynamo* table is created, and the initial template is formed by a simple average of the data folder. The alignment parameters are set up the same way as the previous ones. The alignment project runs quickly, because it only contains 5 particles. The resulting “average of averages” is low-pass filtered, and its contrast is inversed for visualization in Chimera USCF.

#### Block B: Define coordinates of all unit cells

The “average of averages” with the filename result_pr_a_INVERTED.em is opened in Chimera USCF. Using the *volume tracer* tool, the centers of all lattice unit cells are manually marked as shown in Fig 5. The current marker set is then saved with the filename particle_centers.cmm.

#### Block C: Map coordinates back to the averages

Here, the first step of subboxing is performed, where the clicked coordinates are mapped back to each average. First, the resulting table of the alignment project and the clicked coordinates are read into the workspace and prepared to be processed. Then, using the *Dynamo* command dynamo_subboxing_table, the actual subboxing is performed resulting in a temporary *local table* for each tomogram average. This local table contains the unit cell coordinates that are expressed relative to the origin of the tomogram average. Finally, the coordinates are extracted from the local tables and read into a cell array that will be used for the second step of subboxing.

#### Block D: Map coordinates back to the tomograms

**Fig 6:**
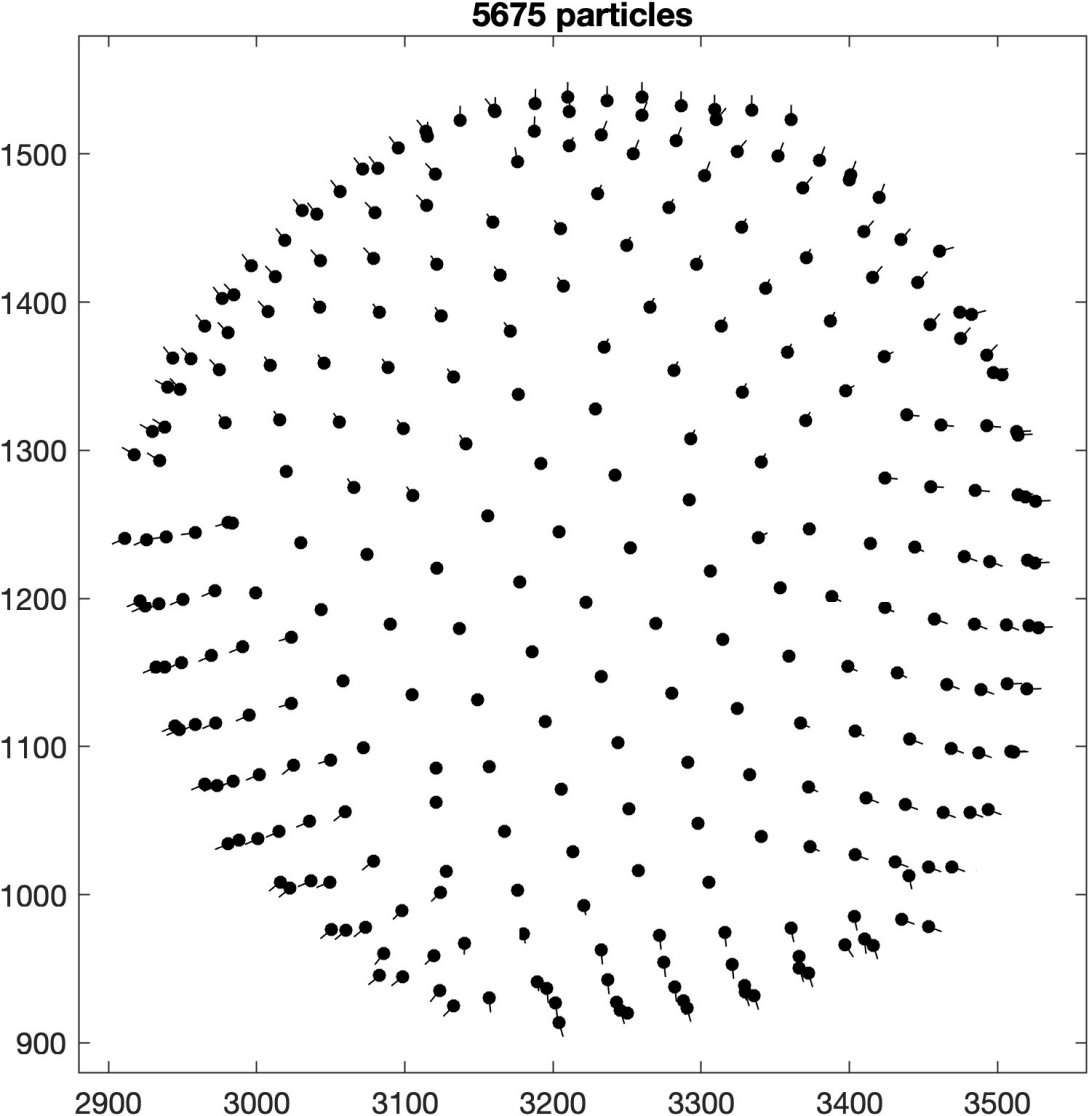
Resulting coordinates from particle picking. Particle coordinates on the surface of one VLP from tomogram *ts001* are shown using the *Dynamo* command dtplot to demonstrate the results from the particle picking. The hexagonal lattice structure inherent to the immature HIV-1 CA-SP1 is clearly visible. A slight deviation from a perfect sphere is also notable. The data and commands to exactly reproduce this figure are available on EMPIAR (EMPIAR-10702). EMPIAR, Electron Microscopy Public Image Archive.

The second step of subboxing is performed here, where the coordinates are mapped from the averages to the tomograms. First, for every tomogram, the previously computed coordinates and the table from the first alignment project are loaded and used in the dynamo_subboxing_table function for subboxing. For each coordinate, a table is then generated that contains the transformed coordinate relative to every subvolumes origin. The tables from all coordinates are merged, resulting in a final table that contains the candidate positions of all lattice units in the tomogram. Due to the previous oversampling, we expect many coordinates to overlap. The table can be corrected for this effect by using the *Dynamo* function dpktbl.exclusionPerVolume that reduces coordinates within a user specified radius to a single coordinate. Coordinates that incidentally describe defect or nonexisting particles will be removed in the next step. The final coordinates are visualized in Fig 6. About 5,000 to 9,000 coordinates per tomogram should be defined. We use those to extract a new set of subvolumes with a side length of 192 pixels, which generously fit a full unit cell of the hexameric lattice. The raw average of these subvolumes (without alignment) will contain some artifacts, since the z-axis orientation of the subboxed subvolumes is inherited from the previous average in which they were picked. This new z-axis does not always coincide with the normal vector of the VLP surface due to its curvature. This effect will disappear in the second alignment project.

#### Block F: Second alignment project

The new particles are aligned in a second alignment project for each tomogram. Because the particle positions are now more accurate, the angular search space and shift limit are reduced compared to the first alignment project. Subvolumes are still binned on the fly, since at this point, high resolution is not of interest yet. The results will be used for the following classification step.

### Step 6: Cross-correlation classification

#### Description

This is the second step of particle picking, in which subvolumes that contain defective particles (or none at all) are automatically removed from the dataset. This is again done for each tomogram separately using cross-correlation (CC) thresholding.

#### Block A: CC threshold

**Fig 7:**
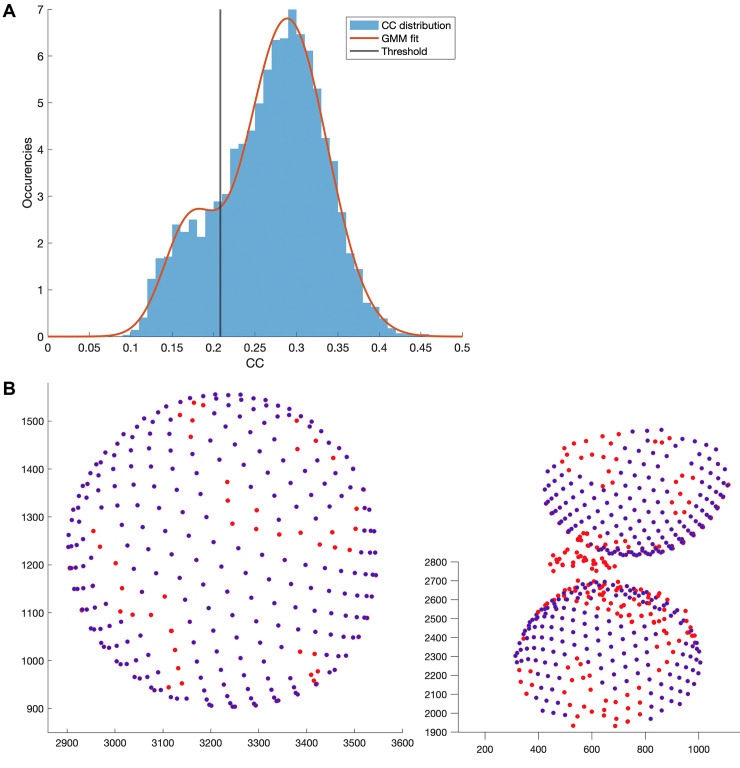
Particle exclusion through CC classification. **(A)** GMM fit to the CC score distribution of the particles from tomogram *ts001* and the automatically defined threshold for particle exclusion. **(B)** Example of excluded particle coordinates (red) of some VLPs from tomogram *ts001*. Particles that do not fit in the expected lattice geometry are excluded. Visualized using the *Dynamo* command dtplot. The data and commands to exactly reproduce this figure are available on EMPIAR (EMPIAR-10702). CC, cross-correlation; EMPIAR, Electron Microscopy Public Image Archive; GMM, Gaussian mixture model; VLP, virus-like particle.

The CC between each aligned subvolume and the final average is stored in the last generated table. A histogram of the CC score shows 2 populations. The population with the lower CC score is assumed to be the “bad” class that needs to be excluded from the processing. It contains mainly particles with particularly bad quality (e.g., stemming from defects) or subvolumes that contain just noise. To automate the exclusion process, a Gaussian mixture model (GMM) is fitted to the CC score distribution. GMMs are commonly used for classification, and the specific one used here describes the combination of 2 Gaussian distributions. Using the MATLAB function fitgmdist, the GMM is fitted to the CC score distribution. The threshold is then defined by taking the minimum between the 2 Gaussian peaks and by adding an empirically defined constant of 0.01. An example of the threshold determination for one tomogram is shown in Fig 7A, and the resulting particle exclusion is visualized in Fig 7B. Coordinates that describe the same particle are again fixed, and a new average for each tomogram is computed.

#### Block B: Adjust height of particles

In the next step, all subvolumes from all tomograms will be combined into one single dataset. Since they have been processed independently so far, the center of their unit cell might vary between tomograms. It therefore needs to be ensured that the unit cells from different tomograms share the same *z*-height inside the subvolume (axis orientation and *x/y*-shifts are already consistent due to the imposed C6 symmetry). This is done by aligning the average of the tomogram to a synthetic reference and then applying the resulting transformation parameters (here just a shift in *z*-direction) to all particles in the corresponding table.

#### Block C: Re-crop

**Fig 8:**
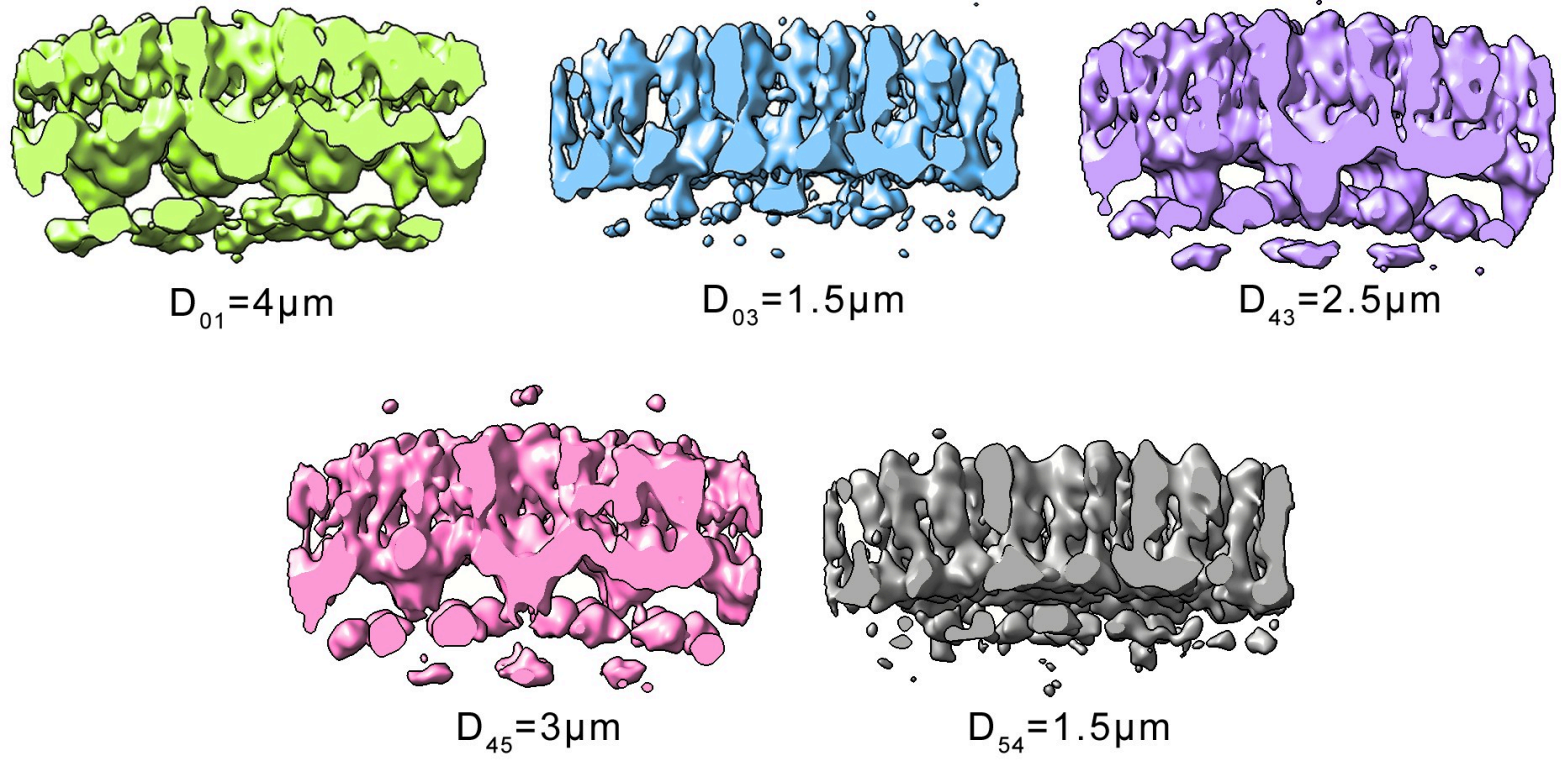
Results of per-tomogram subtomogram averages. Overview of all subtomogram averages from all tomograms (low-pass filtered and cut through center). Structural differences caused by the different D of the tomograms are visible (subscripts refer to tomogram number as stated in Table 1). D, defoci.

The particle picking is completed, and using the coordinates from the last table, all subvolumes are re-extracted one last time (using the previous side length of 192 pixels). About 2,000 to 5,000 particles per tomogram are expected. This dataset now consists of subvolumes that all contain one centered unit cell. The quality of the particles and their initial orientation are good enough to serve as basis for the following gold standard alignment. Fig 8 shows a compilation of all tomogram averages. Note the structural differences caused by the different defoci of the tomograms.

### Step 7: Gold standard alignment

#### Description

Now that all particles have been picked, the gold standard refinement can be started, i.e., the subvolumes from all tomograms are first combined and then split into 2 datasets of the same size that are processed independently in 2 different alignment projects.

#### Block A: Combine tomograms and generate even/odd dataset

First, the tables from all tomograms are merged. Then, using the *star file* functionality of *Dynamo*, the 5 tomogram datasets are combined by creating a single star file that contains the absolute path to all subvolumes. This star file will later serve as data input for the alignment projects. Since the star file also contains the particle tag numbers, the combined table can be used in the conventional way. This method is preferred because it does not require to copy or move any subvolumes from their original location. The combined table is split into a dataset containing the even numbered particle tags and another one containing the odd number ones (about 8,500 particles each). An average for each half-dataset is finally generated that will serve as reference for the following alignment projects.

#### Block B: Set up even/odd projects

The 2 half-datasets are processed in 2 independent alignment projects using the starting references that have been created before. A total of 3 rounds with 3 iterations each are set up. The parameter search space is reduced after each round. The low-pass filter is increased to 32 pixels (8.1 Å), and the last 2 rounds are run on full-sized particles.

### Step 8: Refinement

#### Description

The previous results are refined by pruning again the dataset and running a final alignment project with adapted parameters.

#### Block A: CC filter again (normalized and per tomogram)

To remove particles of low quality, the dataset is again reduced through a CC thresholding. For each half-dataset, the thresholding is done for each tomogram separately since the different defocus values of the tomograms influence the overall CC score (a global CC thresholding might exclude too many particles of a specific defocus value, reducing the coverage of the zeros of the CTF). Subvolumes with a CC score lower than 1 standard deviation below the mean are removed. Prior to computing the threshold, the CC score is normalized for the angle of latitude, since particles at the equator of the VLPs have generally a higher CC score than particles on the poles due to missing wedge effects. The normalization is done with the function dpksta.filters.byCC by fitting the first 2 terms of a general Fourier series to the data. Using the reduced tables, a new average is computed that will serve as reference for the last alignment project.

#### Block B: Refine even/odd projects

Before creating the alignment project, an alignment mask that roughly follows the curvature of the VLPs is defined. The 2 alignment projects are then set up using the same parameters as the last round of the previous project but with a slightly increased low-pass filter (38 fourier pixels corresponding to 6.8 Å) and using the new alignment mask.

### Step 9: Prepare half-maps

#### Description

The 2 resulting averages (half-maps) from the gold standard processing are saved for postprocessing.

#### Block A: Prepare half-maps

The even half-map is aligned to the odd half-map, and the resulting transformation parameters are used to transform the table corresponding to the even half-map. The table is then used to re-average the even particles. This re-averaging is done to avoid edge artifacts caused by the half-map alignment. The 2 half-maps can now be used for the gold standard Fourier shell correlation (FSC) computation. A final map, which will also be used for sharpening, is additionally computed by re-averaging all particles of the full dataset. Here, the re-averaging is necessary to ensure a correct Fourier compensation of the final map (www.dynamo-em.org/w/index.php/Fourier_compensation_during_averaging).

### Postprocessing

**Fig 9:**
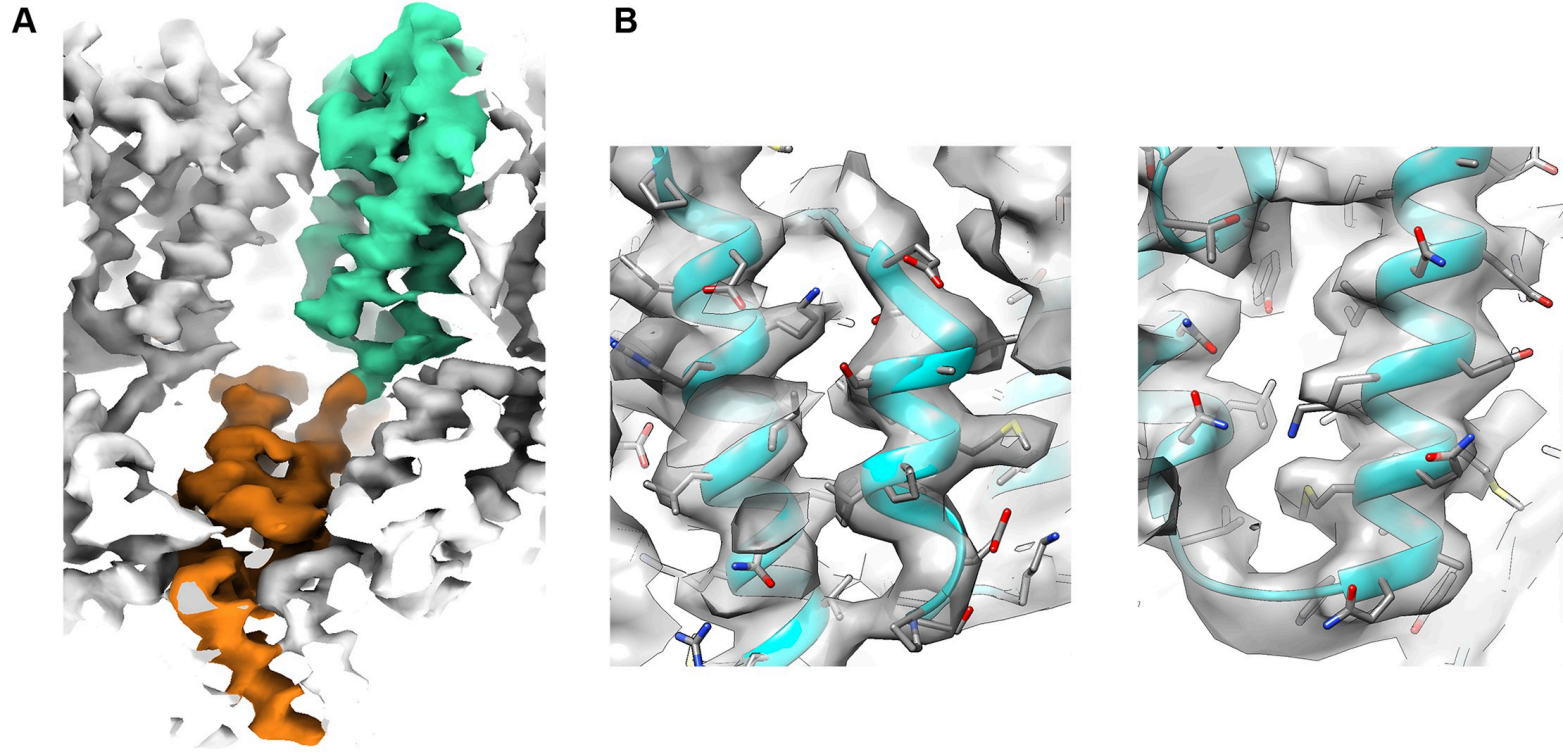
Electron density map after postprocessing. **(A)** A CA-SP1 monomer is shown with highlighted CA-N-terminal domain (orange) and CA-C-terminal domain (green). For an easy comparison of the structures, a similar view to the one shown in [21] is displayed. **(B)** A rigid fit of the corresponding PDB entry 5l93 shows the quality of the map. PDB, Protein Data Bank.

The resolution estimation is done in RELION by computing the mask-corrected FSC curve between the 2 half-maps and using the cutoff criterion of 0.143. The half-maps created in the end are already named in the RELION convention and can therefore directly be loaded into the software. A tight and soft mask is used for the FSC computation. This mask is created by Gaussian filtering and binarizing the final average in an iterative manner until the full central unit cell is included inside the mask and the Gaussian falloff of the mask does not overlap with the structure. Our resolution estimate is 4.5 Å (see FSC curve in S1A Fig). This resolution is identical to the resolution estimated in [21] using the same dataset and 2D CTF correction (Fig 9).

Local resolution is estimated using the blocres function from the Bsoft software package. A box size of 23 pixels can be used with an FSC cutoff criterion of 0.5 and C6 symmetry. The results of local resolution estimation are shown in S1B Fig.

The final density map is generated with the RELION command relion_image_handler using a B-factor of -270, which is slightly stronger compared to the Guinier plot estimate (−240 in our case). The low-pass filter is set to the estimated resolution of 4.5 Å. Finally, the hand of the map is adjusted using the command clip flipyz from IMOD. Details of the final density map including a fit of the molecular structure are shown in S1 Fig. Orthogonal sections of the final average are shown in S1C Fig.

## Conclusions

The goal of this protocol paper was to provide a practical and easily reproducible guide for STA processing to assist new and experienced users in the community. We did this by establishing and documenting a processing pipeline for particle picking and particle alignment in STA. The pipeline is based on the *Dynamo* software package in combination with MATLAB functions. The procedure was applied on the dataset of immature HIV-1 VLPs (EMPIAR-10164), representing a geometry often seen in electron tomography. We validated our pipeline by successfully reproducing the results from [21] that were generated with the same dataset.

By combining and automating key processing tasks and by eliminating redundant steps, we further managed to streamline and automate large parts of the pipeline. The only user interactions are the manual annotations of features in the tomograms and subtomograms. These interactions were specifically designed to minimize the manual effort: Only 2 points per VLP need to be marked, and only 2 particle averages need to be annotated once for centering and once for subboxing. All other steps including classification by CC thresholding were automated. By introducing the 2-step subboxing for particle picking, we provided an alternative way for robust particle coordinate determination. We also proved that using full-sized WBP tomograms from the very beginning of the processing is sufficient for this type of data and that the use of pre-binned tomograms or tomograms with alternative reconstruction methods is not necessary. However, for difficult datasets, e.g., tomograms with more complex geometry, smaller particles, or lower signal-to-noise ratio, we still recommend to start with high contrast reconstruction methods such as, e.g., simultaneous iterative reconstruction technique (SIRT) or similar algorithms. The use of binned tomograms is, however, redundant, as *Dynamo* allows to bin the subvolumes on the fly during their alignment.

We documented the STA pipeline in great detail through this report and through extensive comments in the code itself. We further provided multiple links to additional online guides, documentations, and materials. The processing scripts are also integrated in *Dynamo* where they will be maintained and updated. Users are welcome to submit feedback or suggestions for improvements. We additionally documented all relevant steps and parameters for the pre- and postprocessing to enable users to reproduce the results.

We hope that the provided material will serve as a basis for user-specific projects, benchmarking efforts, or teaching and training purposes. We further encourage the community to publish many protocols and methods for STA processing to support the fast growth of this field.

## Supporting information

S1 Table Computation time for each processing step. Summary of the approximate computation times (walltimes) of each processing step. The times were determined using a configuration of 16–28 CPUs, 32–64 GB RAM, and 6 modern GPUs (variations in CPU and RAM due to changes in available resources from our computing cluster). For the manual interventions (compare Fig 1), we expect users to spend about 10 minutes per tomogram for the definition of dipole models, 5 minutes to define the particle center, and 15 minutes to define the coordinates of all unit cells. CPU, central processing unit; GPU, graphic processing unit; RAM, random access memory.
(PDF)

S2 Table Overview of all files. Summary of all intermediate files that are used or generated during each processing step. The 2 question marks “??” stand for the tomogram numbers 01, 03, 43, 45, and 54 (as stated in Table 1). One question mark “?” stands for the catalogue tomogram numbers (1, 2, 3, 4, and 5). All listed files are available online, expect for the individual particles themselves (these can be directly generated with the command dtcrop using the provided crop tables and tomograms as input). For the alignment project data, only the results are provided. After creating an alignment project with the commands of the processing scripts (without actually running the project), the provided directory results of the corresponding project can simply be copied into the newly generated project folder to skip the processing. For the catalogue, only the geometry models are provided. They can be copied into the catalogue folder after it was generated by the user in order to skip the tomogram annotation (press synchronize models in the catalogue manager after copying the models).
(PDF)

S1 Fig Resolution estimation and final average. **(A)** Mask-corrected FSC curve showing a global resolution estimation of 4.5 angstrom at the 0.143 cutfoff. **(B)** Local resolution estimation (cutoff 0.5) showing variations in resolution across the map. **(C)** Orthogonal slices of the final average. The yellow lines show the positions of the slices. The data and commands to exactly reproduce this figure are available on EMPIAR (EMPIAR-10702). EMPIAR, Electron Microscopy Public Image Archive; FSC, Fourier shell correlation.
(TIF)

S1 Appendix Processing scripts. Processing scripts setup.m, oversample.m, locate.m, and refine.m.
(PDF)

## Abbreviations

CC: cross-correlation
CTF: contrast transfer function
EMPIAR: Electron Microscopy Public Image Archive
FSC: Fourier shell correlation
GMM: Gaussian mixture model
GPU: graphic processing unit
GUI: graphical user interface
PDB: Protein Data Bank
RAM: random access memory
RMS: root mean square
SIRT: simultaneous iterative reconstruction technique
STA: subtomogram averaging
VLP: virus-like particle
WBP: weighted backprojection
