# Supplementary material for: Step-by-step guide to efficient subtomogram averaging of virus-like particles with Dynamo
Source: PLoS Biol. 2021 Aug 26;19(8):e3001318. doi: 10.1371/journal.pbio.3001318 (PMC8389376; doi:10.1371/journal.pbio.3001318)
Supplement: S1 Table — Summary of the approximate computation times (walltimes) of each processing step. The times were determined using a configuration of 16–28 CPUs, 32–64 GB RAM, and 6 modern GPUs (variations in CPU and RAM due to changes in available resources from our computing cluster). For the manual interventions (compare Fig 1), we expect users to spend about 10 minutes per tomogram for the definition of dipole models, 5 minutes to define the particle center, and 15 minutes to define the coordinates of all unit cells. CPU, central processing unit; GPU, graphic processing unit; RAM, random access memory. (PDF) [file pbio.3001318.s001.pdf]

**Processing step**	**Approx. computation time**
Step 1	1min
Step 2	3h
Step 3	2h
Step 4	1.5h per tomogram (total 7.5h)
Step 5, Block A	10min
Step 5, Block C-D	4h
Step 5, Block E	3.5h per tomogram (total 17.5h)
Step 6	5h
Step 7, Block A	1h
Step 7, Block B	48h per half-set (total 96h)
Step 8, Block A	1h
Step 8, Block B	24h per half-set (total 48h)
Step 9	1h
Total processing time	186h
