# Supplementary material for: Step-by-step guide to efficient subtomogram averaging of virus-like particles with Dynamo
Source: PLoS Biol. 2021 Aug 26;19(8):e3001318. doi: 10.1371/journal.pbio.3001318 (PMC8389376; doi:10.1371/journal.pbio.3001318)
Supplement: S2 Table — Summary of all intermediate files that are used or generated during each processing step. The 2 question marks “??” stand for the tomogram numbers 01, 03, 43, 45, and 54 (as stated in Table 1). One question mark “?” stands for the catalogue tomogram numbers (1, 2, 3, 4, and 5). All listed files are available online, expect for the individual particles themselves (these can be directly generated with the command dtcrop using the provided crop tables and tomograms as input). For the alignment project data, only the results are provided. After creating an alignment project with the commands of the processing scripts (without actually running the project), the provided directory results of the corresponding project can simply be copied into the newly generated project folder to skip the processing. For the catalogue, only the geometry models are provided. They can be copied into the catalogue folder after it was generated by the user in order to skip the tomogram annotation (press synchronize models in the catalogue manager after copying the models). (PDF) [file pbio.3001318.s002.pdf]

	Necessary input files		Output files	
**Step**	**File name**	**File type**	**File name**	**File type**
1	b001ts0??.rec	tomogram		
	c001	catalogue		
	tomograms.doc	doc file		
2			c001/tomograms/volume_?/models/mdipoleSet.omd	catalogue model
			pa_ts0??_s256	particle folder
			pa_ts0??_s256/crop.tbl	table
			pa_ts0??_s256/template.em	em map
3	pa_ts001_s256/crop.tbl	table	pa_ts001_s256/crop_RandAz.tbl	table
	pa_ts001_s256	particle folder	pa_ts001_s256/template_RandAz.em	em map
			pr_ts001_0	project
			result_pr_ts001_0_INVERTED.em	em map
			reference_center.cmm	cmm file
			result_pr_ts001_0_recentered.em	em map
4	pa_ts0??_s256/crop.tbl	table	pa_ts0??_s256/crop_RandAz.tbl	table
	result_pr_ts001_0_recentered.em	em map	pr_ts0??_1	project
	pa_ts0??_s256	particle folder		
5	pr_ts0??_1	project	pa_averages_pr1	particle folder
	tomograms.doc	doc file	pa_averages_pr1/crop.tbl	table
			pa_averages_pr1/template.em	em map
			pr_a	project
			result_pr_a_INVERTED.em	em map
			particle_centers.cmm	cmm file
			subbox_ts0??.tbl	table
			pa_ts0??_s192_sb1	particle folder
			pa_ts0??_s192_sb1/crop.tbl	table
			pa_ts0??_s192_sb1/template.em	em map
			pr_ts0??_2	project
6	pr_ts0??_2	project	t_ccFilt_Ex_ts0??.tbl	table
			pa_ts0??_s192_sb1/average_ccFilt_Ex.em	em map
			axis_alignment_template_h.em	em map
			t_ccFilt_Ex_Ali_Ex_ts0??.tbl	table
			pa_ts0??_s192_sb2	particle folder
			pa_ts0??_s192_sb2/crop.tbl	table
			pa_ts0??_s192_sb2/template.em	em map
7	pa_ts0??_s192_sb2	particle folder	merged_particles.star	star file
	pa_ts0??_s192_sb2/crop.tbl	table	referenceE.em	em map
			referenceO.em	em map
			merged_particlesE.tbl	table
			merged_particlesO.tbl	table
			pr_E	project
			pr_O	project
8	pr_E	project	tE_ccGood.tbl	table
	pr_O	project	tO_ccGood.tbl	table
	merged_particles.star	star file	aE_for_refinement.em	em map
	pa_ts0??_s192_sb2	particle folder	aO_for_refinement.em	em map
			mem_mask_merged_adapted.em	em map
			my_smask.em	em map
			pr_E_2	project
			pr_O_2	project
9	pr_E_2	project	half1_final_reaveraged_unfil.mrc	em map
	pr_O_2	project	half2_final_reaveraged_unfil.mrc	em map
	merged_particles.star	star file	final_reaveraged.mrc	em map
	pa_ts0??_s192_sb2	particle folder		
