# Supplementary figures and images for: Step-by-step guide to efficient subtomogram averaging of virus-like particles with Dynamo
Source: PLoS Biol. 2021 Aug 26;19(8):e3001318. doi: 10.1371/journal.pbio.3001318 (PMC8389376; doi:10.1371/journal.pbio.3001318)

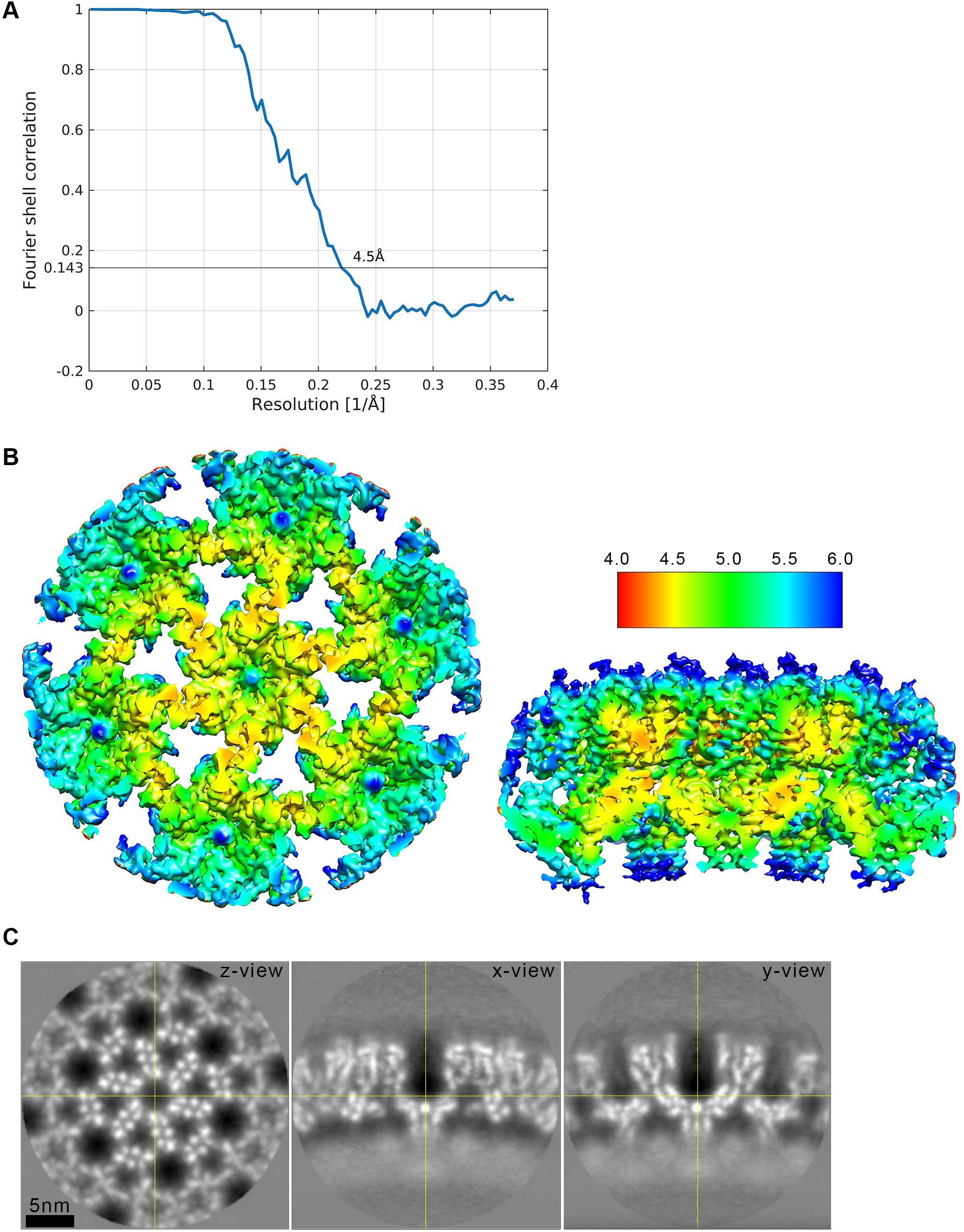

Supplement: S1 Fig — (A) Mask-corrected FSC curve showing a global resolution estimation of 4.5 angstrom at the 0.143 cutfoff. (B) Local resolution estimation (cutoff 0.5) showing variations in resolution across the map. (C) Orthogonal slices of the final average. The yellow lines show the positions of the slices. The data and commands to exactly reproduce this figure are available on EMPIAR (EMPIAR-10702). EMPIAR, Electron Microscopy Public Image Archive; FSC, Fourier shell correlation. (TIF) [file pbio.3001318.s003.tif]
