# Supplementary material for: Step-by-step guide to efficient subtomogram averaging of virus-like particles with Dynamo
Source: PLoS Biol. 2021 Aug 26;19(8):e3001318. doi: 10.1371/journal.pbio.3001318 (PMC8389376; doi:10.1371/journal.pbio.3001318)
Supplement: S1 Appendix — Processing scripts setup.m, oversample.m, locate.m, and refine.m. (PDF) [file pbio.3001318.s004.pdf]

Listing 1: Processing script `setup.m`

```
%%%%%%%%%%%%%%%%%%%%%%%%%%%%%%%%%%%%%%%%%%%%%%%%%%%%%%%%%%%%%%%%  Step 1: Set up inputs

%%%%%%%%%%%%%%%%%%%%%%
%  Block A:  Inputs  %
%%%%%%%%%%%%%%%%%%%%%%
%
% User specific and geometry specific inputs. May need to be adpated.

% catalogue related inputs:
catPath       = '../catalogues/c001';
docFilePath   = '../catalogues/tomograms.doc';

% particle folders path (will contain all particle folders)
particleFolderPath = '../particles/';

% geometry related inputs:
sep           = 120;   % separation in pixel for oversampling of dipole model surfaces
boxSizeFirst  = 256;   % box size for first  alignment project
boxSizeSecond = 192;   % box size for second alignment project
dTh           = 37;    % distance threshold to eliminate particle dublicates

% computation related inputs:
mw            = 16;     % number of matlab workers (CPU cores) for averaging
gpu           = [0:7];  % GPU id's for alignment projects


%%%%%%%%%%%%%%%%%%%%%%%%%%%%%%%%%%%%%%%%%%%%%%%%%
%  Block B:  Parse inputs & generate filenames  %
%%%%%%%%%%%%%%%%%%%%%%%%%%%%%%%%%%%%%%%%%%%%%%%%%
%
% Setting up filenames for automatically generated files. This can be left as it is.

% read and parse doc file
fileID  = fopen(docFilePath); D = textscan(fileID,'%d %s'); fclose(fileID);
tomoID  = D{1,1}';              % get tomogram ID
nTomo   = length(D{1,2});       % get total number of tomograms

% project names
pr_0    = 'pr_ts001_0';         % project to generate reference
pr_a    = 'pr_a';               % alignment project of averages
pr_EO   = {'pr_E'  ,'pr_O'  };  % even/odd projects
pr_EO_2 = {'pr_E_2','pr_O_2'};  % evon/odd projects refinement

% table names
tEO_ccGood_TableName{1} = 'tE_ccGood.tbl';         % cc filtered even for refinement
tEO_ccGood_TableName{2} = 'tO_ccGood.tbl';         % cc filtered odd  for refinement
tableFileNameEO{1}      = 'merged_particlesE.tbl'; % merged particles even
tableFileNameEO{2}      = 'merged_particlesO.tbl'; % merged particles odd
tableFileName           = 'merged_particles.tbl';  % merged particles all

% star file name for merged particles
starFileName    = 'merged_particles.star';

% em maps names
template_name_1 = ['result_' pr_0 '_recentered.em'];
template_name_h = 'axis_alignment_template_h.em';
refFilenameEO   = {'referenceE.em','referenceO.em'};
refEven         = 'aE_for_refinement.em';
refOdd          = 'aO_for_refinement.em';
refMask         = 'mem_mask_merged_adapted.em';

% create tomogram specific strings (idx = array index of tomogram and not tomogram ID)
for idx = 1:nTomo

    % stack name
    stackName{idx} = D{1,2}{idx,1}(end-8:end-4);

```

```
    % particle folders
    targetFolder_1{idx} = [particleFolderPath 'pa_' stackName{idx} '_s256'    ];
    targetFolder_2{idx} = [particleFolderPath 'pa_' stackName{idx} '_s192_sb1'];
    targetFolder_3{idx} = [particleFolderPath 'pa_' stackName{idx} '_s192_sb2'];

    % project names
    pr_1{idx} = ['pr_' stackName{idx} '_1'];
    pr_2{idx} = ['pr_' stackName{idx} '_2'];

end

% folder name for particles of alignment project of averages
folderAllaverages = [particleFolderPath 'pa_averages_pr1'];

% define which particles will be used to create first reference
targetFolder_0 = targetFolder_1{1};
```

Listing 2: Processing script `oversample.m`

```
%%%%%%%%%%%%%%%%%%%%%%%%%%%%%%%%%%%%%%%%  Step 2: Oversampling of VLP hexameric lattice

%%%%%%%%%%%%%%%%%%%%%%%%%%%%%%%%%%%%
%  Block A:  Define dipole models  %
%%%%%%%%%%%%%%%%%%%%%%%%%%%%%%%%%%%%
%
% Manually create dipole models for each VLP through catalogue.
% Do this before running this script.


%%%%%%%%%%%%%%%%%%%%%%%%%%%%%%%%%%%%%
%  Block B:  Process dipole models  %
%%%%%%%%%%%%%%%%%%%%%%%%%%%%%%%%%%%%%
%
% Create crop points on VLP surface (oversampling).

% read dipole models from catalogue into matlab workspace
dcmodels(catPath,'tc','dipoleSet','gm',1,'ws','o');

% loop over tomograms and run model workflow
for idx = 1:nTomo

    % read model
    ds = o.models{idx};

    c=1; tv=[];
    for i=1:length(ds.dipoles)                  % loop over models

        % vesicle workflow
        v = dmodels.vesicle();                  % create empty vesicle model
        v.center = ds.dipoles{i}.center;        % add dipole center to vesicle model
        v.radius = norm( ds.dipoles{i}.north ...
            - ds.dipoles{i}.center);            % add radius
        v.separation = sep;                     % separation of crop points (in px)
        v.crop_distance_from_surface = 0;
        v.updateCrop();                         % update vesicle model

        tv{c} = v.grepTable();                  % create crop table from vesicle model
        tv{c}(:,22) = i;                        % add model number to table

        % check consistency
        if ~strcmp(ds.cvolume.file(end-8 : end-4), stackName{idx})
            warning('CAREFUL: Model index and tomogram ID not consistent.')
        end

        tv{c}(:,20) = tomoID(idx);              % add tomogram number to table
        c=c+1;
    end
```

```
    % merge model specific tables to tomogram specific table and visualize
    tAll = dynamo_table_merge(tv,'linear_tags',1);
    figure; dtplot(tAll,'pf','oriented_positions'); axis equal

    % crop particles
    dtcrop(docFilePath,tAll,targetFolder_1{idx},boxSizeFirst,'mw',mw);
    finalTable = dread([targetFolder_1{idx} '/crop.tbl']);

    % average particles, save in same particle folder and visualize
    oa = daverage(targetFolder_1{idx},'t',finalTable,'fc',1,'mw',mw);
    dwrite(oa.average,[targetFolder_1{idx} '/template.em']);
    dview(oa.average)
end


%%%%%%%%%%%%%%%%%%%%%%%%%%%%%%%%%%%%%%%%%%%%%%%%%%%%%  Step 3: Create initial reference

%%%%%%%%%%%%%%%%%%%%%%%%%%%%%%%%%%%%%%%%%%%%%%%
%  Block A:  Alignment project for reference  %
%%%%%%%%%%%%%%%%%%%%%%%%%%%%%%%%%%%%%%%%%%%%%%%
%
% Generate an initial reference average using one tomogram.

% create first template with randomized azimuth
tOri    = dread([targetFolder_0 '/crop.tbl']);
tRandAz = dynamo_table_randomize_azimuth(tOri);
oa      = daverage(targetFolder_0,'t',tRandAz,'fc',1,'mw',mw);
dwrite(oa.average,[targetFolder_0 '/template_RandAz.em']);
dwrite(tRandAz,   [targetFolder_0 '/crop_RandAz.tbl']);

% create alignment project
dcp.new(pr_0,'d',targetFolder_0,'t',[targetFolder_0 '/crop_RandAz.tbl'], ...
    'template',[targetFolder_0 '/template_RandAz.em'],'masks','default','show',0);

% set alignment parameters for 2 rounds
dvput(pr_0,'ite', [3  3]);    % n iterations
dvput(pr_0,'dim', [64 128]);  % subvolume sidelength (binning)
dvput(pr_0,'low', [23 23]);   % lowpass filtere
dvput(pr_0,'cr',  [60 30]);   % cone range
dvput(pr_0,'cs',  [10 5]);    % cone search step
dvput(pr_0,'ir',  [90 30]);   % inplane rotation
dvput(pr_0,'is',  [10 5]);    % inplane search step
dvput(pr_0,'rf',  [5  5]);    % refinement
dvput(pr_0,'rff', [2  2]);    % refinement factor
dvput(pr_0,'lim', [80 20]);   % shift limit
dvput(pr_0,'limm',[1  2]);    % limit mode
dvput(pr_0,'sym', 'c1');      % symmetry

% set computational parameters
dvput(pr_0,'dst','matlab_gpu','cores',1,'mwa',mw);
dvput(pr_0,'gpus',gpu);

% check/unfold/run
dvrun(pr_0,'check',true,'unfold',true);

% prepare resulting average for chimera
aPath = ddb([pr_0 ':a']);
a = dread(aPath);
dwrite(dynamo_bandpass(a,[1 23])*(-1),['result_' pr_0 '_INVERTED.em']);
```

Listing 3: Processing script `locate.m`

```
%%%%%%%%%%%%%%%%%%%%%%%%%%%%%%%%%%%%%%
%  Block B:  Define particle center  %
%%%%%%%%%%%%%%%%%%%%%%%%%%%%%%%%%%%%%%
%
```

```
% Define manually center of particle in chimera ucsf (for next step).
% Save coordinate as: reference_center.cmm
% Do this before running this script_C.m


%%%%%%%%%%%%%%%%%%%%%%%%%%%%%%%%%%%%%%%%%%%%%%%
%  Block C:  Center particles and re-average  %
%%%%%%%%%%%%%%%%%%%%%%%%%%%%%%%%%%%%%%%%%%%%%%%
%
% Center the initial reference.

% prepare coordinates
cmmFile   = 'reference_center.cmm';                % .cmm file with center coordinate
newCenter = dpcomp.chimera.cmm2mat(cmmFile,1,1);  % read .cmm file
newCenter = round(newCenter);                      % round the coordinates
boxCenter = (boxSizeFirst/2+1) * [1,1,1];         % center of box

% compute vector pointing from box center to new coordinate
rSubunitFromCenter = newCenter - boxCenter;

% get table from project that will be transformed
tOriPath  = ddb([pr_0 ':rt']);
tOri      = dread(tOriPath);

% transform table
T    = dynamo_rigid('shifts',-rSubunitFromCenter);  % create transformation
tSub_1 = dynamo_table_rigid(tOri,T);                  % transform table

% re-average, save and visualize
% this will be the starting reference for the next projects
oa = daverage(targetFolder_0,'t',tSub_1,'fc',1,'mw',mw);
dwrite(oa.average,template_name_1);
dview(oa.average)


%%%%%%%%%%%%%%%%%%%%%%%%%%%%%%%%%%%%%%%%%%%%%%%%%%%%%%  Step 4: First alignment project

%%%%%%%%%%%%%%%%%%%%%%%%%%%%%%%%%%%%%%%
%  Block A:  First alignment project  %
%%%%%%%%%%%%%%%%%%%%%%%%%%%%%%%%%%%%%%%
%
% Run an alignment project for each tomogram.
% Reference is centered final average from previous project.

for idx = 1:nTomo

    % randomize azimuth of table before using it in project
    tOri    = dread([targetFolder_1{idx} '/crop.tbl']);
    tRandAz = dynamo_table_randomize_azimuth(tOri);
    dwrite(tRandAz, [targetFolder_1{idx} '/crop_RandAz.tbl'])

    % define first alignment project
    dcp.new(pr_1{idx},'d',targetFolder_1{idx}, ...
        't',[targetFolder_1{idx} '/crop_RandAz.tbl'],'template', template_name_1, ...
        'masks','default','show',0);

    % set alignment parameters for 2 rounds
    dvput(pr_1{idx},'ite', [3  3]);
    dvput(pr_1{idx},'dim', [64 128]);
    dvput(pr_1{idx},'low', [23 23]);
    dvput(pr_1{idx},'cr',  [60 30]);
    dvput(pr_1{idx},'cs',  [10 5]);
    dvput(pr_1{idx},'ir',  [90 30]);
    dvput(pr_1{idx},'is',  [10 5]);
    dvput(pr_1{idx},'rf',  [5  5]);
    dvput(pr_1{idx},'rff', [2  2]);
    dvput(pr_1{idx},'lim', [80 20]);
    dvput(pr_1{idx},'limm',[1  2]);
```

```
    dvput(pr_1{idx},'sym', 'c6');    % from now on c6 symmetry

    % set computational parameters
    dvput(pr_1{idx},'dst','matlab_gpu','cores',1,'mwa',mw);
    dvput(pr_1{idx},'gpus',gpu);

    % check/unfold/run
    dvrun(pr_1{idx},'check',true,'unfold',true);

    % check resulting table with visualization in plot
    tPath = ddb([pr_1{idx} ':rt']);
    t = dread(tPath);
    figure; dtplot(t,'pf','oriented_positions'); axis equal
    %dpktbl.plots.disks(t,'r',boxSizeFirst/2)

end


%%%%%%%%%%%%%%%%%%%%%%%%%%%%%%%%%%%%%%%%%%%%%%%%  Step 5: Determine candidate particles

%%%%%%%%%%%%%%%%%%%%%%%%%%%%%%%%%%%%%%%%%%%%
%  Block A:  Create 'average of averages'  %
%%%%%%%%%%%%%%%%%%%%%%%%%%%%%%%%%%%%%%%%%%%%
%
% Align averages from first alignment project to prepare first step of subboxing.

% create folder for previous averages
mkdir(folderAllaverages)

% copy previous averages into new folder (particle tag number = tomogram number)
for idx = 1:nTomo
    aPath = ddb([pr_1{idx} ':a']);
    copyfile(aPath{1}, ...
        [folderAllaverages '/particle_' num2str(tomoID(idx),'%06.f') '.em';])
end

% create corresponding table and save it in new particle folder
ta          = dynamo_table_blank(nTomo);
ta(:,13)    = 0;                        % no missing wedge compensation needed
ta(:,20)    = ta(:,1);                  % set particle tag number = tomogram number
ta(:,24:26) = 129*ones(nTomo,3);        % set centers
dwrite(ta,[folderAllaverages '/crop.tbl'])

% create template, put it in particle folder and visualize
oa = daverage(folderAllaverages,'t',[folderAllaverages '/crop.tbl'],'fc',1);
dwrite(oa.average,[folderAllaverages '/template.em']);
dview(oa.average) % visualize

% create the alignment project of averages to align the 5 particles (averages)
dcp.new(pr_a,'d',folderAllaverages,'t',[folderAllaverages '/crop.tbl'], ...
    'template',[folderAllaverages '/template.em'],'masks','default','show',0);

% set alignment parameters for 2 rounds
dvput(pr_a,'ite', [3  3]);
dvput(pr_a,'dim', [64 128]);
dvput(pr_a,'low', [23 23]);
dvput(pr_a,'cr',  [60 30]);
dvput(pr_a,'cs',  [10 5]);
dvput(pr_a,'ir',  [90 30]);
dvput(pr_a,'is',  [10 5]);
dvput(pr_a,'rf',  [5  5]);
dvput(pr_a,'rff', [2  2]);
dvput(pr_a,'lim', [80 20]);
dvput(pr_a,'limm',[1  2]);
dvput(pr_a,'sym', 'c6');

% set computational parameters
dvput(pr_a,'dst','matlab_gpu','cores',1,'mwa',mw);
```

```
dvput(pr_a,'gpus',gpu);

% check/unfold/run
dvrun(pr_a,'check',true,'unfold',true);

% prepare resulting average for chimera
aPath = ddb([pr_a ':a']);
a = dread(aPath);
dwrite(dynamo_bandpass(a,[1 23])*(-1),['result_' pr_a '_INVERTED.em']);
```

Listing 4: Processing script `refine.m`

```
%%%%%%%%%%%%%%%%%%%%%%%%%%%%%%%%%%%%%%%%%%%%%%%%%%%%
%  Block B:  Define coordinates of all unit cells  %
%%%%%%%%%%%%%%%%%%%%%%%%%%%%%%%%%%%%%%%%%%%%%%%%%%%%
%
% Define manually center of all unit cells in chimera (for next step).
% Save coordinate as: particle_centers.cmm
% Do this before running this script_D.m


%%%%%%%%%%%%%%%%%%%%%%%%%%%%%%%%%%%%%%%%%%%%%%%%%%%%
%  Block C:  Map coordinates back to the averages  %
%%%%%%%%%%%%%%%%%%%%%%%%%%%%%%%%%%%%%%%%%%%%%%%%%%%%
%
% First step of subboxing, where the coordinates are mapped back onto the averages.

% get table from the alignment project of averages
tOriPathCombined = ddb([pr_a ':rt']);
tOriCombined = dread(tOriPathCombined);

% prepare coordinates
cmmFileCombined   = 'particle_centers.cmm';         % .cmm file with center coordinates
newCenterCombined = dpcomp.chimera.cmm2mat(cmmFileCombined,1,1); % read .cmm file
newCenterCombined = round(newCenterCombined);       % round the coordinates
boxCenterCombined = (boxSizeFirst/2+1) * [1,1,1];   % center of box

% compute vector from box center to new coordinate for each coordinate
% and create mini table for each average that contains the corresponding coordinates
for i=1:length(newCenterCombined(:,1))
    disp(['working on point: ' num2str(i)])
    rSubunitFromCenterCombined(i,:) = newCenterCombined(i,:) - boxCenterCombined;
    tSubCombined{i} = ...
        dynamo_subboxing_table(tOriCombined,rSubunitFromCenterCombined(i,:));
end

% merge mini tables
tSubAllCombined = dynamo_table_merge(tSubCombined,'linear_tags',1);

% create the newCenter variable for each average that will be used in next steps
for idx = 1:nTomo
    tSubOneCombined    = tSubAllCombined(tSubAllCombined(:,20)==tomoID(idx),:);
    newCenterCell{idx} = tSubOneCombined(:,24:26);
end


%%%%%%%%%%%%%%%%%%%%%%%%%%%%%%%%%%%%%%%%%%%%%%%%%%%%%
%  Block D:  Map coordinates back to the tomograms  %
%%%%%%%%%%%%%%%%%%%%%%%%%%%%%%%%%%%%%%%%%%%%%%%%%%%%%
%
% Second step of subboxing, where coordinates are mapped back into the tomograms.

% subbox each tomogram
for idx = 1:nTomo

    % get table from first alignment
    tOriPath = ddb([pr_1{idx} ':rt']);
```

```
    tOri = dread(tOriPath);

    % prepare coordinates
    newCenter = newCenterCell{tomoID(idx)};     % get centers from subboxing
    newCenter = round(newCenter);               % round the coordinates
    boxCenter = (boxSizeFirst/2+1) * [1, 1, 1]; % center of box

    % compute vector from box center to new subbox center and perform subboxing
    for i=1:length(newCenter(:,1))
        disp(['working on: ' num2str(i)])
        rSubunitFromCenter(i,:) = newCenter(i,:) - boxCenter;
        tSub{i} = dynamo_subboxing_table(tOri,rSubunitFromCenter(i,:));
    end

    % merge tables
    tSubAll = dynamo_table_merge(tSub,'linear_tags',1);

    % exclude coordinates describing the same unit cell
    tSubAllEx = dpktbl.exclusionPerVolume(tSubAll,dTh);

    % plot and save table
    figure; h = dpktbl.plots.sketch(tSubAllEx,'haxis',gca()); h.zlength.value=100;
    dwrite(tSubAllEx,['subbox_' stackName{idx} '.tbl'])
end

% crop particles for each tomogram
for idx = 1:nTomo

    % re-crop subboxed particles
    tSubAllEx = dread(['subbox_' stackName{idx} '.tbl']);
    dtcrop(docFilePath,tSubAllEx,targetFolder_2{idx},boxSizeSecond,'mw',mw);

    % re-average subboxed particles
    finalTable = dread([targetFolder_2{idx} '/crop.tbl']);
    oa = daverage(targetFolder_2{idx},'t',finalTable,'fc',1,'mw',mw);
    dwrite(oa.average,[targetFolder_2{idx} '/template.em']);
    dview(oa.average)
end


%%%%%%%%%%%%%%%%%%%%%%%%%%%%%%%%%%%%%%%%
%  Block E:  Second alignment project  %
%%%%%%%%%%%%%%%%%%%%%%%%%%%%%%%%%%%%%%%%
%
% run second alignment project with new table and template

for idx = 1:nTomo

    % set up project
    dcp.new(pr_2{idx},'d',targetFolder_2{idx}, ...
        't',[targetFolder_2{idx} '/crop.tbl'], ...
        'template', [targetFolder_2{idx} '/template.em'], ...
        'masks','default','show',0);

    % set alignment parameters for 1 round with 3 iterations
    dvput(pr_2{idx},'ite', 3);
    dvput(pr_2{idx},'dim', 96);
    dvput(pr_2{idx},'low', 23);
    dvput(pr_2{idx},'cr',  45);
    dvput(pr_2{idx},'cs',  5);
    dvput(pr_2{idx},'ir',  30);
    dvput(pr_2{idx},'is',  5);
    dvput(pr_2{idx},'rf',  4);
    dvput(pr_2{idx},'rff', 2);
    dvput(pr_2{idx},'lim', [30,30,30]);
    dvput(pr_2{idx},'limm',3);
    dvput(pr_2{idx},'sym','c6');

```

```matlab
    % set up computational parameters
    dvput(pr_2{idx},'dst','matlab_gpu','cores',1,'mwa',mw);
    dvput(pr_2{idx},'gpus',gpu);

    % check/unfold/run
    dvrun(pr_2{idx},'check',true,'unfold',true);
end


%%%%%%%%%%%%%%%%%%%%%%%%%%%%%%%%%%%%%%%%%%%%%  Step 6: Cross-correlation classification

%%%%%%%%%%%%%%%%%%%%%%%%%%%
%  Block A:  CC theshold  %
%%%%%%%%%%%%%%%%%%%%%%%%%%%
%
% Automatically exclude false-positive subvolumes by cc thresholding.

for idx = 1:nTomo

    % get table
    tPath = ddb([pr_2{idx} ':rt']);
    t = dread(tPath);

    % fit gaussian mixture model (GMM) to cc distribution
    gmdist  = fitgmdist(t(:,10), 2);
    gmsigma = gmdist.Sigma;
    gmmu    = gmdist.mu;

    % define threshold at minimum between gaussians plus constant
    x      = 0:0.001:0.5;
    TF     = islocalmin(pdf(gmdist, x'));
    thresh = x(TF)'+0.01;

    % use std in case no threshold found
    [maxMu, maxMuI] = max(gmmu(:));
    if isempty(thresh)
       thresh = maxMu - 1.5*sqrt(gmsigma(maxMuI));
    end

    % plot results of fit
    figure;
    hold on;
    histogram(t(:,10), 'Normalization', 'pdf', 'EdgeColor', 'none')
    plot(x, pdf(gmdist, x'))
    xline(thresh)
    xlim([0 0.5])
    legend('CC distribution','GMM fit','Threshold')
    title(['Stack: ' stackName{idx}])
    xlabel('CC')
    ylabel('Occurencies')

    % visualize kept/excluded particles
    figure;
    hold on
    h = dpktbl.plots.sketch(t(t(:,10)>thresh,:),'haxis',gca());
    h.centerSettings.colorFill = 'b';
    h = dpktbl.plots.sketch(t(t(:,10)<thresh,:),'haxis',gca());
    h.centerSettings.colorFill = 'r';
    title(['Particles excluded by CC filtering, stack: ' stackName{idx}])

    % define cc filtered table
    t_ccFilt = t(t(:,10)>thresh,:);

    % Exclude coordinates describing the same unit cell
    t_ccFilt_Ex = dpktbl.exclusionPerVolume(t_ccFilt,dTh);
    dwrite(t_ccFilt_Ex,['t_ccFilt_Ex_' stackName{idx} '.tbl']);
end
```

```
% make new average and save it
for idx = 1:nTomo
    t_ccFilt_Ex = dread(['t_ccFilt_Ex_' stackName{idx} '.tbl']);
    oa = daverage(targetFolder_2{idx},'t',t_ccFilt_Ex,'fc',1,'mw',mw);
    dwrite(oa.average,[targetFolder_2{idx} '/average_ccFilt_Ex.em']);
    dview(oa.average)
end


%%%%%%%%%%%%%%%%%%%%%%%%%%%%%%%%%%%%%%%%%%
%  Block B:  Adjust height of particles  %
%%%%%%%%%%%%%%%%%%%%%%%%%%%%%%%%%%%%%%%%%%
%
% Create consistent height of particles across all tomograms.

% create synthetic reference for alignemnt to height
mr = dpktomo.examples.motiveTypes.Membrane();   % create membrane object
mr.thickness  = 104;                % choose thickness of membrane
mr.radius     = 348;                % choose radius of membrane
mr.sidelength = 192;                % choose sidelength of box
mr.getMask();                       % compute mask
template_h  = (mr.mask)*(-1)+1;     % invert contrast
dwrite(template_h,template_name_h)
dview(template_h)

for idx = 1:nTomo

    % read average
    template_average = dread([targetFolder_2{idx} '/average_ccFilt_Ex.em']);

    % align average to template (only z-shift allowed)
    sal = dalign(dynamo_bandpass(template_average,[1 23]), ...
        dynamo_bandpass(template_h,[1 23]),'cr',1,'cs',1,'ir',1,'is',1,'dim',96, ...
        'limm',1,'lim',[1,1,20],'rf',5,'rff',2);
    dview(sal.aligned_particle);

    % apply transformation to table
    t_ccFilt_Ex      = dread(['t_ccFilt_Ex_' stackName{idx} '.tbl']);
    t_ccFilt_Ex_Ali =  dynamo_table_rigid(t_ccFilt_Ex,sal.Tp);

    % Exclude again coordinates describing the same unit cell
    t_ccFilt_Ex_Ali_Ex = dpktbl.exclusionPerVolume(t_ccFilt_Ex_Ali,dTh);
    dwrite(t_ccFilt_Ex_Ali_Ex, ['t_ccFilt_Ex_Ali_Ex_' stackName{idx} '.tbl']);
end


%%%%%%%%%%%%%%%%%%%%%%%
%  Block C:  Re-crop  %
%%%%%%%%%%%%%%%%%%%%%%%
%
% Extract subvolumes using latest table.

for idx = 1:nTomo
    % read table and re-crop particles
    t_ccFilt_Ex_Ali_Ex = dread(['t_ccFilt_Ex_Ali_Ex_' stackName{idx} '.tbl']);
    dtcrop(docFilePath,t_ccFilt_Ex_Ali_Ex,targetFolder_3{idx},192,'mw',mw);
end

for idx = 1:nTomo
    % Make and save average of last particle folder for sanity check
    finalTable = dread([targetFolder_3{idx} '/crop.tbl']);
    oa = daverage(targetFolder_3{idx},'t',finalTable,'fc',1,'mw',mw);
    dwrite(oa.average,[targetFolder_3{idx} '/template.em']);
    dview(oa.average)
end


%%%%%%%%%%%%%%%%%%%%%%%%%%%%%%%%%%%%%%%%%%%%%%%%%%%%%%  Step 7: Gold standard alignment
```

```
%%%%%%%%%%%%%%%%%%%%%%%%%%%%%%%%%%%%%%%%%%%%%%%%%%%%%%%%%%%%%%%
%  Block A:  Combine tomograms and generate even/odd dataset  %
%%%%%%%%%%%%%%%%%%%%%%%%%%%%%%%%%%%%%%%%%%%%%%%%%%%%%%%%%%%%%%%
%
% Combine all particles from all tomograms and then split them in an even/odd dataset

% create table array
for idx = 1:nTomo
    tableName{idx} = [targetFolder_3{idx} '/crop.tbl'];
end

% create ParticleListFile object (this object only exists temporarily in matlab)
plfClean = dpkdata.containers.ParticleListFile.mergeDataFolders( ...
    targetFolder_3,'tables',tableName);

% create and write the .star file
plfClean.writeFile(starFileName)

% create merged table
tMergedClean = plfClean.metadata.table.getClassicalTable();

% write full/even/odd merged tables
dwrite(tMergedClean,tableFileName)
dwrite(tMergedClean(2:2:end,:),tableFileNameEO{1})
dwrite(tMergedClean(1:2:end,:),tableFileNameEO{2})

% create and save even/odd references
oaE = daverage(starFileName,'t',tableFileNameEO{1},'fc',1,'mw',mw);
oaO = daverage(starFileName,'t',tableFileNameEO{2},'fc',1,'mw',mw);
dwrite(oaE.average,refFilenameEO{1});
dwrite(oaO.average,refFilenameEO{2});


%%%%%%%%%%%%%%%%%%%%%%%%%%%%%%%%%%%%%%%
%  Block B:  Set up even/odd project  %
%%%%%%%%%%%%%%%%%%%%%%%%%%%%%%%%%%%%%%%
%
% Run first even/odd alignemnt projects.

for r = 1:2 % loop over even/odd

    % define alignment project
    dcp.new(pr_EO{r},'t',tableFileNameEO{r},'d',targetFolder_3{1}, ...
        'template',refFilenameEO{r},'show',0,'masks','default');
    dvput(pr_EO{r},'data',starFileName)

    % set alignment parameters for 3 rounds
    dvput(pr_EO{r},'ite', [3  3   3]);
    dvput(pr_EO{r},'dim', [96 192 192]);
    dvput(pr_EO{r},'low', [23 28  32]);
    dvput(pr_EO{r},'cr',  [45 12  6]);
    dvput(pr_EO{r},'cs',  [5  2   1]);
    dvput(pr_EO{r},'ir',  [30 12  6]);
    dvput(pr_EO{r},'is',  [5  2   1]);
    dvput(pr_EO{r},'rf',  [5  4   3]);
    dvput(pr_EO{r},'rff' ,[2  2   2]);
    dvput(pr_EO{r},'lim', [30 12  6]);
    dvput(pr_EO{r},'limm',[2  2   2]);
    dvput(pr_EO{r},'sym','c6')

    % set computational parameters
    dvput(pr_EO{r},'dst','matlab_gpu','cores',1,'mwa',mw);
    dvput(pr_EO{r},'gpus',gpu);

    % check/unfold/run
    dvrun(pr_EO{r},'check',true,'unfold',true);
end
```

```matlab


%%%%%%%%%%%%%%%%%%%%%%%%%%%%%%%%%%%%%%%%%%%%%%%%%%%%%%%%%%%%%%%%%%%  Step 8: Refinement

%%%%%%%%%%%%%%%%%%%%%%%%%%%%%%%%%%%%%%%%%%%%%%%%%%%%%%%%%%%
%  Block A:  CC filter again (normalized & per tomogram)  %
%%%%%%%%%%%%%%%%%%%%%%%%%%%%%%%%%%%%%%%%%%%%%%%%%%%%%%%%%%%
%
% Before the refinemet we exclude partciles with low CC again

% get last tables
tEPath = ddb([pr_EO{1} ':rt']);
tOPath = ddb([pr_EO{2} ':rt']);
tE    = dread(tEPath);
tO    = dread(tOPath);

% remove particles per tomogram based on normalized CC
for idx = 1:nTomo
    [tE_ccGood_Array{idx}, tE_ccBad_Array{idx}] = ...
        dpksta.filters.byCC(tE( tE(:,20)==idx ,:));
    [tO_ccGood_Array{idx}, tO_ccBad_Array{idx}] = ...
        dpksta.filters.byCC(tO( tO(:,20)==idx ,:));
end

% merge tables of accepted particles
tEO_ccGood{1} = dynamo_table_merge(tE_ccGood_Array);
tEO_ccGood{2} = dynamo_table_merge(tO_ccGood_Array);

% save tables
dwrite(tEO_ccGood{1},tEO_ccGood_TableName{1});
dwrite(tEO_ccGood{2},tEO_ccGood_TableName{2});

% make averages for new references and save them
oaE = daverage(starFileName,'t',tEO_ccGood_TableName{1},'fc',1,'mw',mw);
oaO = daverage(starFileName,'t',tEO_ccGood_TableName{2},'fc',1,'mw',mw);
dwrite(oaE.average,refEven);
dwrite(oaO.average,refOdd);


%%%%%%%%%%%%%%%%%%%%%%%%%%%%%%%%%%%%%%%
%  Block B:  Refine even/odd project  %
%%%%%%%%%%%%%%%%%%%%%%%%%%%%%%%%%%%%%%%
%
% Last even/odd alignment project. With alignment mask and stricter lowpass.

% create adapted mask and visualize it
mr = dpktomo.examples.motiveTypes.Membrane();
mr.thickness  = 145;
mr.sidelength = 192;
mr.radius     = 348;
mr.shifts     = [0 0 5];
mr.getMask();
mem_mask  = mr.mask;
cyl_mask = dynamo_cylinder(87,192,[97 97 97]);
final_mask = mem_mask.*cyl_mask;           % subtract cylinder
dwrite(final_mask,refMask)
dwrite(final_mask,'my_smask.em')
dview(final_mask)

% read averages (for template)
aEOPath{1} = refEven;
aEOPath{2} = refOdd;

for r = 1:2

    % define alignment project
    dcp.new(pr_EO_2{r},'t',tEO_ccGood_TableName{r},'d',targetFolder_3{1}, ...
        'template',aEOPath{1,r} ,'show',0,'masks','default');
```

```
    dvput(pr_EO_2{r},'data',starFileName)
    dvput(pr_EO_2{r},'file_mask',refMask)

    % set alignment parameters for 1 round
    dvput(pr_EO_2{r},'ite_r1', 3);
    dvput(pr_EO_2{r},'dim_r1', 192);
    dvput(pr_EO_2{r},'low_r1', 38);
    dvput(pr_EO_2{r},'cr_r1',  6);
    dvput(pr_EO_2{r},'cs_r1',  1);
    dvput(pr_EO_2{r},'ir_r1',  6);
    dvput(pr_EO_2{r},'is_r1',  1);
    dvput(pr_EO_2{r},'rf_r1',  3);
    dvput(pr_EO_2{r},'rff_r1', 2);
    dvput(pr_EO_2{r},'lim_r1', [6,6,6]);
    dvput(pr_EO_2{r},'limm_r1',2);
    dvput(pr_EO_2{r},'sym_r1','c6'); % symmetry introduced

    % set computational parameters
    dvput(pr_EO_2{r},'dst','matlab_gpu','cores',1,'mwa',mw);
    dvput(pr_EO_2{r},'gpus',gpu);

    % check/unfold/run
    dvrun(pr_EO_2{r},'check',true,'unfold',true);
end


%%%%%%%%%%%%%%%%%%%%%%%%%%%%%%%%%%%%%%%%%%%%%%%%%%%%%%%%%%%%%  Step 9: Prepare half-maps

%%%%%%%%%%%%%%%%%%%%%%%%%%%%%%%%%
%  Block A:  Prepare half-maps  %
%%%%%%%%%%%%%%%%%%%%%%%%%%%%%%%%%
%
% Align half-maps and save them for post-processing.

% get last maps
aEPath = ddb([pr_EO_2{1} ':a']);
aOPath = ddb([pr_EO_2{2} ':a']);
aE = dread(aEPath);
aO = dread(aOPath);

% get last tables
tEPath = ddb([pr_EO_2{1} ':rt']);
tOPath = ddb([pr_EO_2{2} ':rt']);
tE     = dread(tEPath);
tO     = dread(tOPath);

% align even to odd (with C6 imposed)
sal = dalign(dynamo_csym(aE,6),dynamo_csym(aO,6),'cr',3,'cs',1,'ir',3,'is',1, ...
    'dim',192,'limm',1,'lim',[3,3,3],'rf',5,'rff',2, ...
    'destination','matlab_gpu','gpu_identifier_set',1);

% transform even table (of which halfmap was aligned)
tEr    = dynamo_table_rigid(tE,sal.Tp);

% make average
oaEr = daverage(starFileName,'t',tEr,'fc',1,'mw',mw);

% save aligned halfmaps (with symmetry) for FSC estimation
dwrite(dynamo_csym(oaEr.average,6) * (-1), 'half1_final_reaveraged_unfil.mrc')
dwrite(dynamo_csym(aO          ,6) * (-1), 'half2_final_reaveraged_unfil.mrc')

% reaverage all particles for post-processing (correct fourier compensation)
oAll = daverage(starFileName,'t',dynamo_table_merge({tEr,tO}),'fc',1,'mw',mw);
dwrite(dynamo_csym(oAll.average,6) * (-1), 'final_reaveraged.mrc')
```
